# A subset of plasma membrane-localized PP2C.D phosphatases negatively regulate SAUR-mediated cell expansion in Arabidopsis

**DOI:** 10.1371/journal.pgen.1007455

**Published:** 2018-06-13

**Authors:** Hong Ren, Mee Yeon Park, Angela K. Spartz, Jeh Haur Wong, William M. Gray

**Affiliations:** Department of Plant and Microbial Biology, University of Minnesota, Saint Paul, Minnesota, United States of America; Wake Forest University, UNITED STATES

## Abstract

The plant hormone auxin regulates numerous growth and developmental processes throughout the plant life cycle. One major function of auxin in plant growth and development is the regulation of cell expansion. Our previous studies have shown that SMALL AUXIN UP RNA (SAUR) proteins promote auxin-induced cell expansion via an acid growth mechanism. These proteins inhibit the PP2C.D family phosphatases to activate plasma membrane (PM) H^+^-ATPases and thereby promote cell expansion. However, the functions of individual PP2C.D phosphatases are poorly understood. Here, we investigated PP2C.D-mediated control of cell expansion and other aspects of plant growth and development. The nine PP2C.D family members exhibit distinct subcellular localization patterns. Our genetic findings demonstrate that the three plasma membrane-localized members, PP2C.D2, PP2C.D5, and PP2C.D6, are the major regulators of cell expansion. These phosphatases physically interact with SAUR19 and PM H^+^-ATPases, and inhibit cell expansion by dephosphorylating the penultimate threonine of PM H^+^-ATPases. *PP2C*.*D* genes are broadly expressed and are crucial for diverse plant growth and developmental processes, including apical hook development, phototropism, and organ growth. *GFP-SAUR19* overexpression suppresses the growth defects conferred by *PP2C*.*D5* overexpression, indicating that SAUR proteins antagonize the growth inhibition conferred by the plasma membrane-localized PP2C.D phosphatases. Auxin and high temperature upregulate the expression of some *PP2C*.*D* family members, which may provide an additional layer of regulation to prevent plant overgrowth. Our findings provide novel insights into auxin-induced cell expansion, and provide crucial loss-of-function genetic support for SAUR-PP2C.D regulatory modules controlling key aspects of plant growth.

## Author summary

The plant hormone auxin is a major regulator of cell expansion, which is a fundamental cellular process essential for plant growth and development. The acid growth theory was proposed in the 1970s to explain auxin-induced cell expansion. However, the mechanistic basis of auxin-induced cell expansion via acid growth is poorly understood. Here, we investigated the functions of the D-clade PP2C (PP2C.D) family phosphatases in auxin-induced cell expansion as well as plant growth and development. The PP2C.D protein family is composed of nine members. Our findings demonstrate that the plasma membrane-localized PP2C.D2, PP2C.D5, and PP2C.D6 family members are the major regulators in auxin-induced cell expansion. These proteins physically associate with SAUR proteins and plasma membrane H^+^-ATPases to negatively regulate cell expansion. *PP2C*.*D* genes are broadly expressed and are crucial for a variety of plant growth and developmental processes, particularly elongation growth, such as hypocotyl and stamen filament growth. The results of our studies provide novel insights into auxin-induced cell expansion via an acid growth mechanism.

## Introduction

The plant hormone auxin regulates nearly all aspects of growth and development, including embryogenesis [1], root development [2], gravitropism [3], leaf development [4], vascular development [5], phototropism [6], shade avoidance [6], shoot apical meristem development [7], flower primordium formation [5], stamen development [8], and gynoecium development [9]. At the cellular level, auxin regulates these processes through the control of cell division, expansion, and differentiation [10, 11]. Auxin is perceived by a co-receptor complex that is composed of TRANSPORT INHIBITOR RESPONSE 1/AUXIN SIGNALING F-BOX PROTEINS (TIR1/AFBs) and AUXIN/INDOLE-3-ACETIC ACID (AUX/IAA) transcriptional repressors in the nucleus. Auxin binding of the TIR1/AFB and AUX/IAA complex leads to the degradation of AUX/IAA proteins by the 26S proteasome [12]. AUX/IAA degradation then relieves the repression of AUXIN RESPONSE FACTOR (ARF) transcription factors to activate the expression of auxin-responsive genes [13]. These auxin-responsive genes, including *SMALL AUXIN UP RNAs* (*SAURs*) [14], then regulate auxin-mediated cellular, physiological, and developmental processes.

A major function of auxin in plant growth and development is the regulation of cell expansion. Auxin-induced cell expansion was hypothesized to occur via an acid growth mechanism, which was first proposed in the 1970s [15, 16]. According to this theory, auxin activates plasma membrane (PM) H^+^-ATPases (known as AHAs/ARABIDOPSIS H+-ATPases in Arabidopsis), which pump protons across the plasma membrane, thus acidifying the apoplast and elevating membrane potential. The more acidic apoplastic pH activates expansins and other cell wall remodeling enzymes, resulting in increased cell wall extensibility. Additionally, plasma membrane hyperpolarization promotes increased solute and water uptake, providing elevated turgor to drive cell expansion. In recent years, several studies have provided molecular support for auxin-mediated PM H^+^-ATPase activation during hypocotyl growth in Arabidopsis. Auxin induces the phosphorylation of the penultimate threonine (Thr^947^ in AHA2), a key regulatory site of PM H^+^-ATPases without altering AHA protein abundance [17]. This increase in AHA Thr^947^ phosphorylation is likely the result of auxin-induced *SAUR* expression, as *GFP-SAUR19* overexpression promotes AHA-Thr^947^ phosphorylation, hypocotyl elongation, and cell wall extensibility [18, 19]. Furthermore, consistent with the hypothesis that acid growth requires auxin-mediated gene expression, the canonical TIR1/AFB-AUX/IAA nuclear signaling pathway was recently shown to be required for auxin-induced hypocotyl elongation and cell wall acidification [20, 21]. Together, these findings provide strong genetic and biochemical evidence in support of the acid growth theory.

SAUR proteins promote PM H^+^-ATPase activation and the resulting cell expansion by inhibiting the activity of type 2C protein phosphatases belonging to the D subfamily (PP2C.D) [18, 19]. PP2Cs are Mg^2+^/Mn^2+^-dependent enzymes that are evolutionarily conserved from prokaryotes to eukaryotes [22, 23]. The Arabidopsis genome encodes eighty PP2Cs, nine of which belong to the D-subclade [22]. Recent studies have implicated PP2C.D family members in the regulation of apical hook development [18, 24], auxin-induced cell expansion [18], leaf senescence [25], and immune response [26]. In our previous work [18], we found that plants harboring an artificial microRNA (amiRNA) targeting five D-clade family members conferred phenotypes similar to, albeit much weaker than, *GFP-SAUR19* overexpression lines, including increased hypocotyl length, hypersensitivity to LiCl, and increased medium acidification. While this finding provided initial genetic support for an antagonistic role of SAUR and PP2C.D proteins in regulating PM H^+^-ATPase activity and cell expansion, the identities and functional relationships of the specific PP2C.D family members involved remained uncertain. Furthermore, the amiRNA reverse genetic approach does not completely abolish gene function and can be prone to off-targeting effects. Here, we extend our studies on the functions of these phosphatases by conducting a genetic characterization of *pp2c*.*d* family loss-of function mutants. We find that the PP2C.D family members exhibit distinct subcellular localization patterns, and the plasma membrane-localized subset, PP2C.D2, D5, and D6 phosphatases, play a major role in antagonizing SAUR-mediated regulation of PM H^+^-ATPase activity, cell expansion, and plant growth and development.

## Results

### *PP2C*.*D* genes are broadly expressed

**Fig 1:**
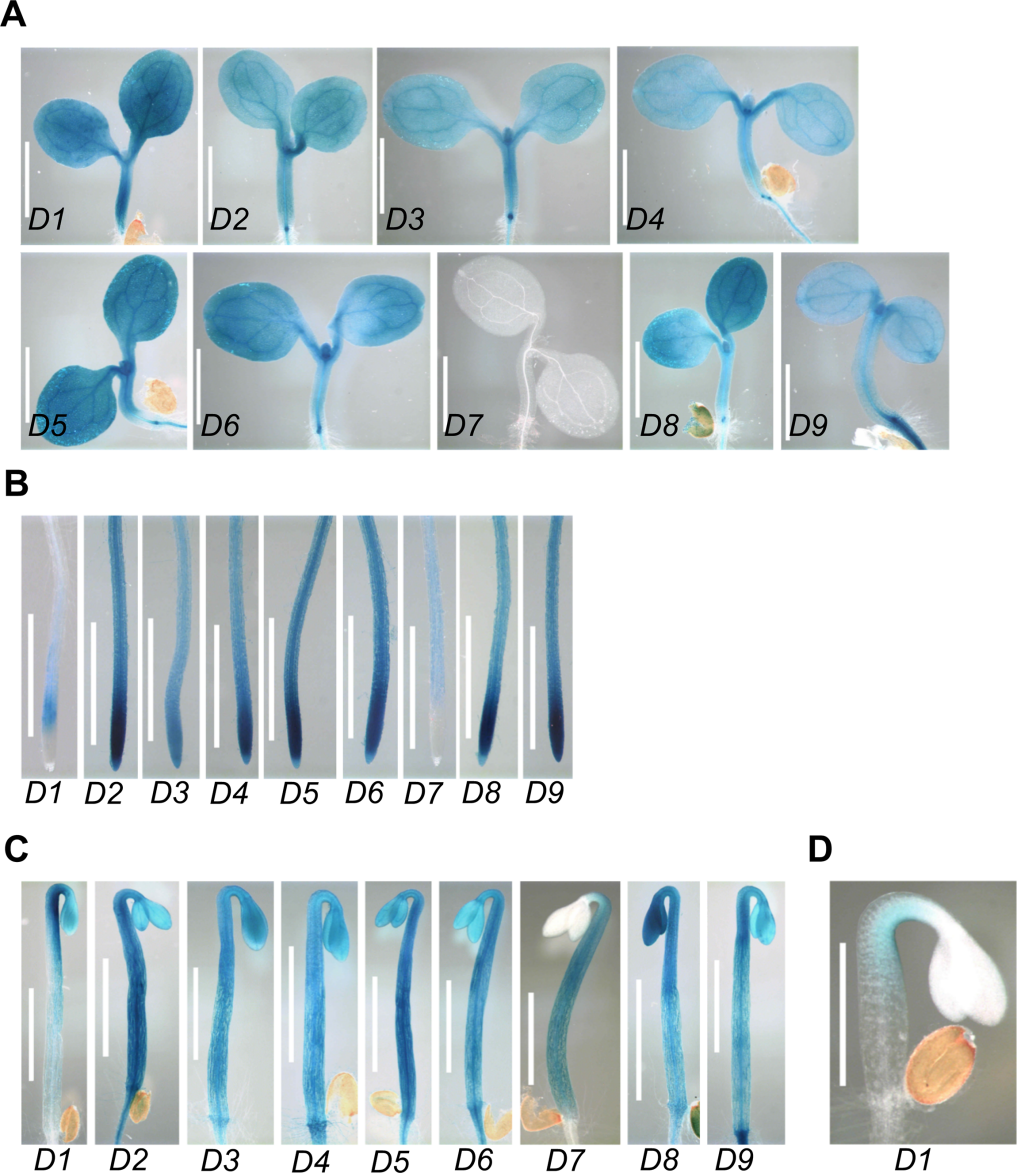
Expression patterns of *PP2C*.*D-GUS* reporters. **(**A) Shoots of 5-day-old light-grown seedlings. (B) Roots of 5-day-old light-grown seedlings. (C) Shoots of 3-day-old etiolated seedlings. (D) Apical hook of a 2-day-old etiolated seedling. GUS staining was performed at 37 ^o^C for 24 (A-C) or 4 h (D). Scale bars = 1 mm (A-C) or 0.5 mm (D).

To investigate the roles of the *PP2C*.*D* family genes in plant growth and development, we examined *PP2C*.*D* gene expression using the *GUS* (β-glucuronidase) reporter gene driven by the native *PP2C*.*D* promoters. We generated Arabidopsis transgenic plants expressing *PP2C*.*D1pro*:*EGFP-GUS* transcriptional or *PP2C*.*D(2–9)pro*:*PP2C*.*D(2–9)-GUS* translational reporter constructs. All *PP2C*.*D* genes except *PP2C*.*D7* were expressed in the cotyledons and hypocotyls of light-grown seedlings (Fig 1A). In the roots of light-grown seedlings, all *PP2C*.*D* genes except *PP2C*.*D1* and *PP2C*.*D7* were ubiquitously expressed (Fig 1B). *PP2C*.*D1* was weakly expressed specifically in the root elongation zone, while *PP2C*.*D7* was weakly expressed throughout the root except for the root tip region (Fig 1B). All *PP2C*.*D* genes were also expressed in the cotyledons and hypocotyls of etiolated seedlings except for *PP2C*.*D7*, which was only expressed in the hypocotyls (Fig 1C). Interestingly, *PP2C*.*D1* exhibited stronger expression on the inner side of the apical hook (Fig 1C and 1D), consistent with previous findings implicating *PP2C*.*D1* in apical hook formation [18, 24]. All *PP2C*.*D* genes except *PP2C*.*D7* were expressed in the petioles and rosette leaves of 2-week-old plants, while *PP2C*.*D7* was only very weakly expressed in the petioles (S1A Fig). In flowers and siliques, all *PP2C*.*D* genes except *PP2C*.*D7* were expressed in multiple floral organs and siliques, while *PP2C*.*D7* was only weakly expressed in petals (S1B and S1C Fig). In particular, *PP2C*.*D1*, *D2*, *D5*, and *D6* were strongly expressed in stamen filaments. *PP2C*.*D8* expression was highly enriched in pistils, and *PP2C*.*D4* and *D6* exhibited strong expression in anthers. The absence or low level of *PP2C*.*D7* expression in nearly all organs examined is consistent with the results of numerous transcriptomic studies compiled in the Arabidopsis eFP Browser database (S2 Fig) [27].

Together, our *GUS* expression data demonstrate that all *PP2C*.*D* genes except *PP2C*.*D7* are broadly expressed in most organs and tissues during the life cycle of plants, especially in the growing organs and tissues, including hypocotyls, young roots and leaves, and stamen filaments. These results indicate that the *PP2C*.*D* family genes may play an important role in a variety of plant growth and developmental processes.

### PP2C.D proteins exhibit distinct subcellular localization

The subcellular localization of the PP2C.D family phosphatases was previously examined using green fluorescent protein (GFP) as a reporter in Arabidopsis transgenic plants harboring reporter constructs driven by the cauliflower mosaic virus (CaMV) *35S* promoter [28]. However, since overexpression driven by the *35S* promoter may cause fluorescent fusion protein mislocalization, we further examined the subcellular localization of the PP2C.D family phosphatases using the native *PP2C*.*D* promoters to express PP2C.D-GFP fusion proteins. We generated *PP2C*.*D(1–9)pro*:*PP2C*.*D(1–9)-GFP* Arabidopsis transgenic plants. Since our GUS reporter analysis indicated that all PP2C.D family members except *PP2C*.*D1* and *D7* were strongly expressed in root tips (Fig 1B), we initially examined PP2C.D-GFP localization in cells of the root meristem and elongation zone.

**Fig 2:**
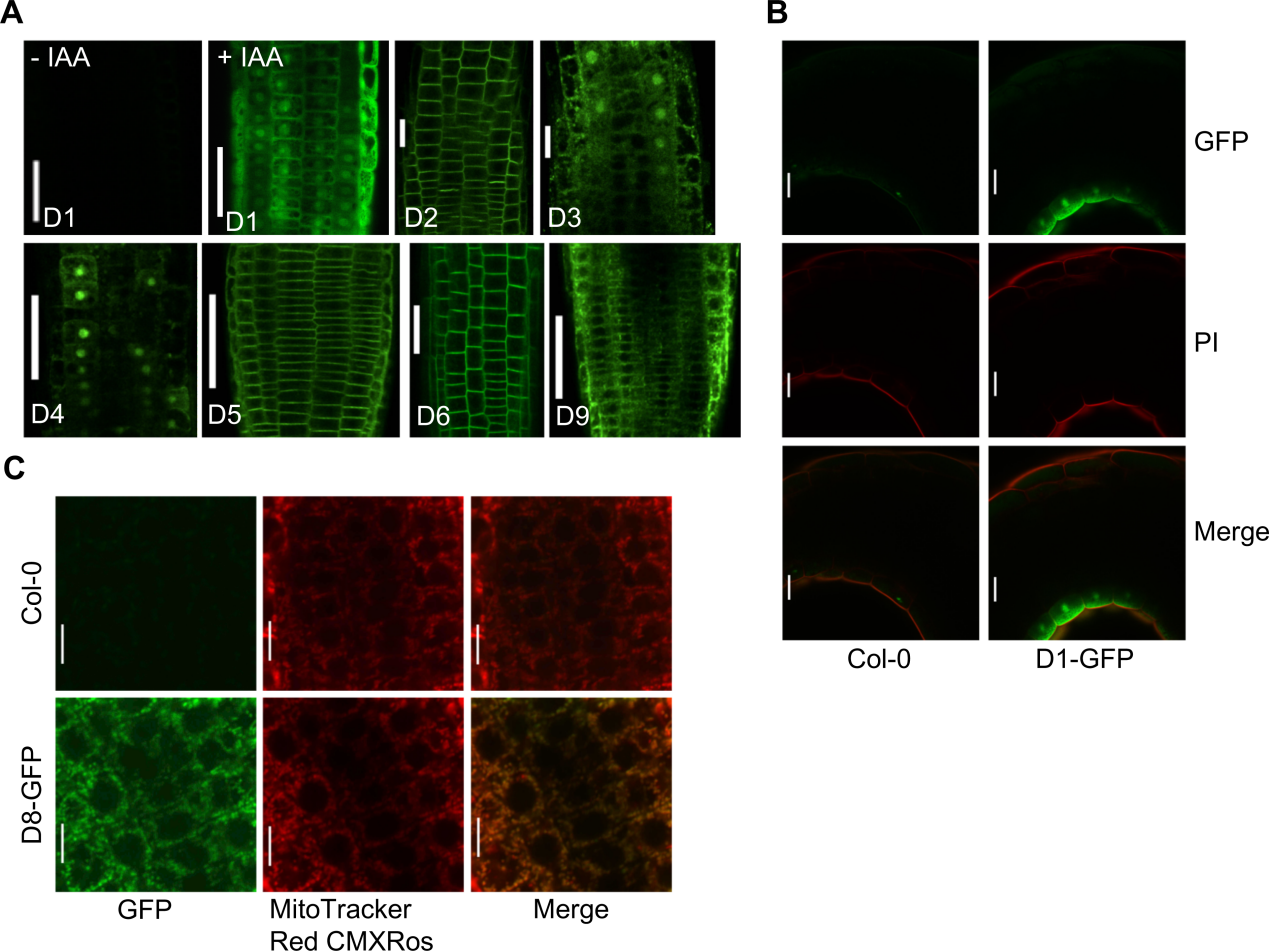
Differential localization of PP2C.D-GFP fusion proteins. (A) Localization of PP2C.D-GFP fusion proteins in the root tips of 5-day-old seedlings. *PP2C*.*D1-GFP* seedlings were treated with 10 μM IAA for 4 h to increase expression to detectable levels. (B) Localization of PP2C.D1-GFP fusion protein in the apical hooks of 2-day-old etiolated seedlings counter-stained with 10 μg/ml propidium iodide (PI) for 30 min. (C) Localization of PP2C.D8-GFP fusion protein in the root tips of 5-day-old seedlings. Seedlings were counter-stained with 0.5 μM MitoTracker Red CMXRos (Invitrogen) for 20 min. (A—C) Root tips and apical hooks were observed under a Nikon A1 spectral confocal microscope. Scale bars = 50 μm (A), 25 μm (B), or 10 μm (C).

Previous studies reported that PP2C.D1, also known as APD7 (Arabidopsis PP2C clade D 7) [28] or SSPP (SENESCENCE-SUPPRESSED PROTEIN PHOSPHATASE) [25], resided in the nucleus and cytoplasm of root cells [28] or only in the cytoplasm of mesophyll protoplasts [25]. Consistent with our *PP2C*.*D1pro*:*EGFP-GUS* reporter findings, PP2C.D1-GFP fluorescence in root tips was below our detection limit. However, since *PP2C*.*D1* is auxin-inducible [29], when *PP2C*.*D1pro*:*PP2C*.*D1-GFP* seedlings were treated with IAA, robust expression was observed, with GFP fluorescence evident in both nuclei and the cytosol (Fig 2A). To better examine PP2C.D1 localization without the complications of exogenous IAA, we examined apical hooks of etiolated seedlings. Consistent with the *GUS* expression pattern (Fig 1C and 1D), GFP fluorescence was specifically observed in the epidermal layer of the inner side of the hook (Fig 2B). Similar to auxin-treated roots, PP2C.D1 appeared to localize to both nuclei and the cytoplasm.

Consistent with previous findings using *35S*-driven GFP reporters [28], PP2C.D2/APD2, PP2C.D5/APD6, and PP2C.D6/APD3 localized exclusively to the cell periphery of root cells, consistent with a plasma membrane localization (Fig 2A). PP2C.D3/PP2C38 was recently reported to reside at the plasma membrane and in intracellular punctae of Arabidopsis leaf epidermal cells overexpressing *PP2C*.*D3-GFP* driven by the *35S* promoter [26]. In contrast to these results, while we did detect some PP2C.D3-GFP signal near the cell periphery, considerable fluorescence was also observed in intracellular punctae and nuclei (Fig 2A). Likewise, PP2C.D4/APD4 exhibited both nuclear and cytosolic localization (Fig 2A). PP2C.D7/APD9 was reported to associate with the plasma membrane and endomembranes [28]. Although we examined many independent *PP2C*.*D7pro*:*PP2C*.*D7-GFP* lines, we could not successfully detect PP2C.D7-GFP protein expression in any organs examined, consistent with the low expression observed with the *GUS* reporter (Fig 1B) and transcriptomic studies (S2 Fig). PP2C.D8-GFP and MitoTracker signals colocalized in root cells (Fig 2C), supporting the previous findings that PP2C.D8/APD5 is localized in the mitochondria [28]. Lastly, Tovar-Mendez et al. (2014) reported that PP2C.D9/APD8 localized to the cytoplasm, but not in the nucleus [28]. Our results support this conclusion, although similar to PP2C.D3, cytosolic fluorescence was frequently punctate rather than uniform (Fig 2A). While we cannot be certain that all PP2C.D-GFP proteins are functional and localize precisely like the endogenous protein, we provide evidence below that the PP2C.D2-, D5-, and D6-GFP constructs encode functional proteins (S5C Fig). Given the high degree of sequence similarity between PP2C.D family members, it seems likely that the addition of a C-terminal GFP tag does not interfere with PP2C.D function. Together, our results indicate that the PP2C.D family phosphatases exhibit distinct subcellular localization, residing in various cellular compartments.

### The plasma membrane-localized subset of PP2C.D proteins inhibit cell expansion

The Arabidopsis hypocotyl is an excellent system to study elongation growth, since its size is mainly controlled by cell expansion [30]. Our previous studies using an amiRNA to knock down multiple members (*PP2C*.*D2*, *D5*, *D7*, *D8*, *D9*) of the *PP2C*.*D* family genes showed that the PP2C.D family phosphatases may function redundantly to negatively regulate hypocotyl growth [18]. The resulting growth phenotypes, however, were weak in comparison to *GFP-SAUR19* overexpression plants. To definitively determine the contribution of individual PP2C.D phosphatases to cell expansion, we analyzed the hypocotyl growth phenotype of all *pp2c*.*d* T-DNA insertion mutants (S3 Fig). Semiquantitative RT-PCR analysis confirmed that the *pp2c*.*d1*, *d2*, *d5*, *d6*, *d7*, *d8*, and *d9* insertion mutants were likely null alleles (S4 Fig). Likewise, the *pp2c*.*d3* and *d4* insertion mutants were previously reported to be null or severe knockdown mutants [26]. *pp2c*.*d5* was the only single mutant that exhibited slightly increased hypocotyl growth (S5A Fig). The *pp2c*.*d4* T-DNA insertion mutant was unavailable when we started to generate various *pp2c*.*d* higher order mutants. The *pp2c*.*d3/4* double mutant was recently published [26], and the mutant seedlings did not exhibit an obvious hypocotyl growth phenotype (S5B Fig).

**Fig 3:**
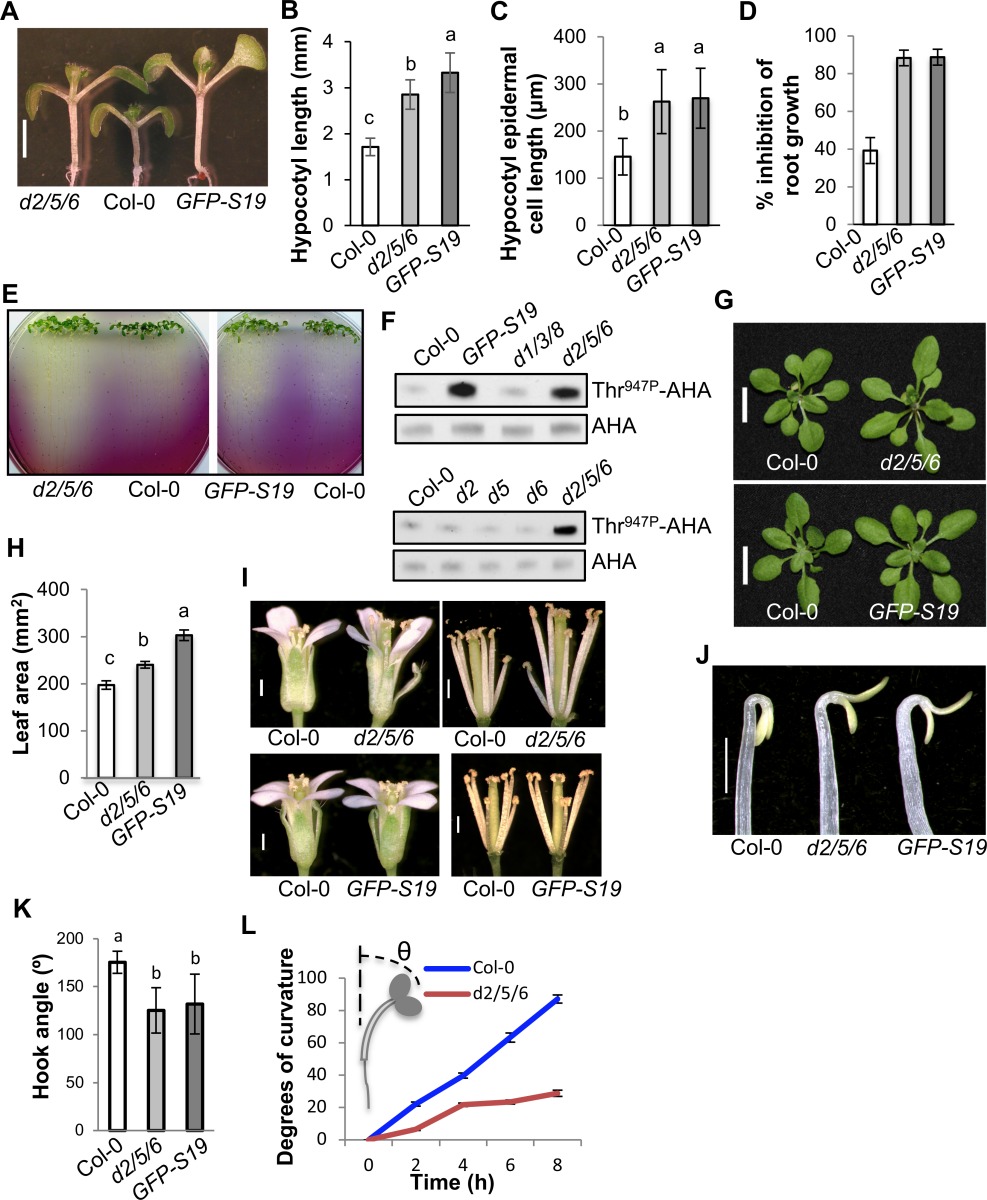
The *pp2c*.*d2/5/6* triple mutant exhibits increased cell expansion and plant growth. (A) Eight-day-old light-grown seedlings. Scale bar = 2 mm. (B) Hypocotyl length of 8-day-old light-grown seedlings. Error bars = SD (n = 47). (C) Epidermal cell length of 8-day-old light-grown seedling hypocotyls. The apical most 10 cells from 10 seedlings were measured. Error bars = SD. (D) LiCl root inhibition assays. Five-day-old light-grown seedlings grown on ATS plates were transferred onto ATS or ATS + 10 mM LiCl plates for 3 days. New root growth after transfer was measured. Error bars = SD (n = 25–38). (E) Medium acidification assays. Six-day-old light-grown seedlings grown on ATS plates were transferred onto plates containing the pH indicator bromocresol purple (BCP, pH6.5), and the medium color change was observed 6 days later. **(**F) Levels of Thr^947^-phosphorylated AHA proteins as monitored by GST-14-3-3 binding. Five micrograms of microsomal fractions prepared from 6-day-old light-grown seedlings were loaded. AHA and GST-14-3-3-bound AHA proteins were detected by anti-AHA and anti-GST antibodies, respectively. (G) Shoots of 3-week-old plants. Scale bar = 1 cm. (H) Rosette leaf areas of 3-week-old plants. Total leaf areas of the plants were measured using Photoshop. Error bars = SEM (n = 23–28). (I) Flowers. Scale bars = 0.5 mm. (J) Apical hooks of 3-day-old etiolated seedlings. Scale bar = 0.5 mm. (K) Apical hook angles of 3-day-old etiolated seedlings. Error bars = SD (n = 29–35). (L) Reduced phototropic growth of *pp2c*.*d2/5/6* seedlings. Four-day-old etiolated seedlings were photo-stimulated with unilateral blue light for 2, 4, 6, and 8 h, and the angles of hypocotyl bending were measured by ImageJ. Error bars = SEM (n = 14–16). (B, C, H, and K) Different letters above the bars indicate significant differences (P < 0.05).

The lack of strong single mutant phenotypes suggested functional redundancy within the *PP2C*.*D* gene family. We therefore generated a variety of double, triple, and quadruple mutants (S5A Fig). Given the plasma membrane localization of SAUR19 and PM H^+^-ATPases, we were particularly interested in lines that lack the three plasma membrane-localized family members, PP2C.D2, D5, and D6. Interestingly, mutations in *PP2C*.*D2* or *PP2C*.*D6* enhanced the hypocotyl growth phenotype of *pp2c*.*d5* (S5A Fig). Likewise, the *pp2c*.*d2 pp2c*.*d6* double mutant exhibited a long hypocotyl phenotype. Hypocotyl length was unaffected, however, in all of the other double mutant combinations tested. Furthermore, the *pp2c*.*d2/5/6* triple mutant seedlings displayed an even stronger hypocotyl growth phenotype, exhibiting hypocotyls nearly as long as those of *GFP-SAUR19* overexpression seedlings (Figs 3A, 3B and S5A). Like *GFP-SAUR19* seedlings, this increase in hypocotyl length was the result of increased cell expansion (Fig 3C). In contrast, various combinations of triple mutants for *pp2c*.*d1*, *d3*, *d8*, and *d9* did not exhibit any obvious hypocotyl growth phenotype (S5A Fig). We previously found that *PP2C*.*D1* overexpression under the control of the *35S* promoter conferred a dramatic reduction in hypocotyl length and plant stature [18]. Loss-of-function analysis, however, indicates that endogenous *PP2C*.*D1* plays little if any role in hypocotyl elongation under our growth conditions, as the *pp2c*.*d1* mutation failed to enhance the hypocotyl growth phenotype of the *pp2c*.*d2/5/6* triple mutant as well as lower order mutant combinations (S5A Fig).

To confirm that the *pp2c*.*d2/5/6* long hypocotyl phenotype was in fact due to loss of the three PM-localized phosphatases, we transformed the triple mutant with the *PP2C*.*D(2*,*5*,*6)pro*:*PP2C*.*D(2*,*5*,*6)-GFP* reporter constructs used to assess localization (Fig 2). All three GFP fusion constructs restored hypocotyl length to at least the corresponding double mutant (S5C Fig). In the *D2-* and *D5-GFP* lines, over-complementation was observed, with hypocotyl lengths returning wild-type length. Presumably, this is due to position effects that may result in modest *PP2C*.*D* overexpression. Together, the above genetic findings indicate that the plasma membrane-localized PP2C.D2, D5, and D6 proteins are the major PP2C.D phosphatases that negatively regulate cell expansion during hypocotyl growth.

Since the *pp2c*.*d2/5/6* triple mutant exhibited a long hypocotyl phenotype, we proceeded to assess this mutant for other *GFP-SAUR19* overexpression-related phenotypes. Like *GFP-SAUR19* seedlings, *pp2c*.*d2/5/6* seedlings exhibited dramatic hypersensitivity to 10 mM LiCl (Fig 3D) and increased medium acidification (Fig 3E), phenotypes suggestive of elevated PM H^+^-ATPase activity [18, 31]. To test this possibility, we examined AHA-Thr^947^ phosphorylation status indirectly using a GST-14-3-3 far western blotting assay. Several prior studies have demonstrated that this assay accurately reflects AHA-Thr^947^ phosphorylation status and the corresponding changes in PM H^+^-ATPase activity [17, 18, 32, 33]. A striking increase in AHA-Thr^947^ phosphorylation was observed in both *GFP-SAUR19* and *pp2c*.*d2/5/6* seedlings (Fig 3F). In contrast, AHA-Thr^947^ phosphorylation levels in the *pp2c*.*d1/3/8* triple mutant were not noticeably different from wild-type (Fig 3F). The strong genetic interaction observed in hypocotyl growth assays suggested that PP2C.D2, D5, and D6 act in a redundant fashion (S5A Fig). Consistent with this notion, while the *pp2c*.*d2/5/6* triple mutant exhibited increased AHA-Thr^947^ phosphorylation levels, phosphorylation in the single mutants was comparable to wild-type (Fig 3F). Together, our genetic and biochemical findings demonstrate that these PM-localized PP2C.D family members function redundantly to regulate PM H^+^-ATPase activity to control cell expansion.

Although *pp2c*.*d2/5/6* plants exhibited no major developmental abnormalities, several growth phenotypes were apparent in older plants. Three-week-old *pp2c*.*d2/5/6* plants exhibited slightly larger rosette leaves than those of wild-type, but similar to the case for hypocotyl growth, this phenotype was slightly more dramatic in *GFP-SAUR19* plants (Fig 3G and 3H). In flowers, *pp2c*.*d2/5/6* flowers exhibited longer stamen filaments and pistils than those of wild-type flowers (Fig 3I). While *GFP-SAUR19* flowers did not exhibit long stamen filament and pistil phenotypes (Fig 3I), increased stamen filament length has been reported for plants expressing SAUR63-GFP or GUS fusion proteins [34]. Plant height and silique length of mature *pp2c*.*d2/5/6* plants were also slightly larger than wild-type (S5D and S5E Fig). While *GFP-SAUR19* plants did not exhibit an obvious silique growth phenotype (S5E Fig), increased silique growth was recently reported in transgenic Arabidopsis plants overexpressing *SAUR8*, *SAUR10*, and *SAUR16* [35].

*PP2C*.*D1* is differentially expressed in the apical hook of etiolated seedlings (Figs 1D and 2B), and etiolated *pp2c*.*d1* mutants, as well as *GFP-SAUR19* seedlings, exhibit defective apical hook development [18, 24]. While the *pp2c*.*d2*, *d5*, and *d6* single mutants develop apical hooks comparable to wild-type [18], given the functional redundancy we observed in other assays of these family members, we examined the apical hook phenotype of the *pp2c*.*d2/5/6* triple mutant. Indeed, like etiolated *GFP-SAUR19* seedlings, *pp2c*.*d2/5/6* seedlings exhibited partially opened apical hooks and expanded cotyledons (Fig 3J and 3K). In our previous work [36], *GFP-SAUR19* seedlings were shown to exhibit reduced phototropism, suggesting the involvement of SAUR proteins in tropic growth responses. Consistent with this notion, *SAUR* transcripts have been found to preferentially accumulate on the elongating side of bending organs [37–39]. We therefore examined whether PP2C.D2, D5, and D6 may function in phototropic response. When exposed to unilateral blue light, etiolated *pp2c*.*d2/5/6* seedlings exhibited dramatically reduced phototropic curvature (Fig 3L), suggesting that SAUR-mediated inhibition of PP2C.D2/5/6 activity on the light distal side of the hypocotyl may underlie phototropic bending. Based on the increased growth phenotypes of *pp2c*.*d2/5/6* plants and their strong phenotypic similarity to *SAUR* gain-of-function plants, as well as similar effects on PM H^+^-ATPase Thr^947^ phosphorylation, our results suggest that PP2C.D2, D5, and D6 phosphatases are the primary effectors of plasma membrane-localized SAUR proteins that regulate plant growth.

### PP2C.D phosphatases interact with SAUR19 and PM H^+^-ATPases

**Fig 4:**
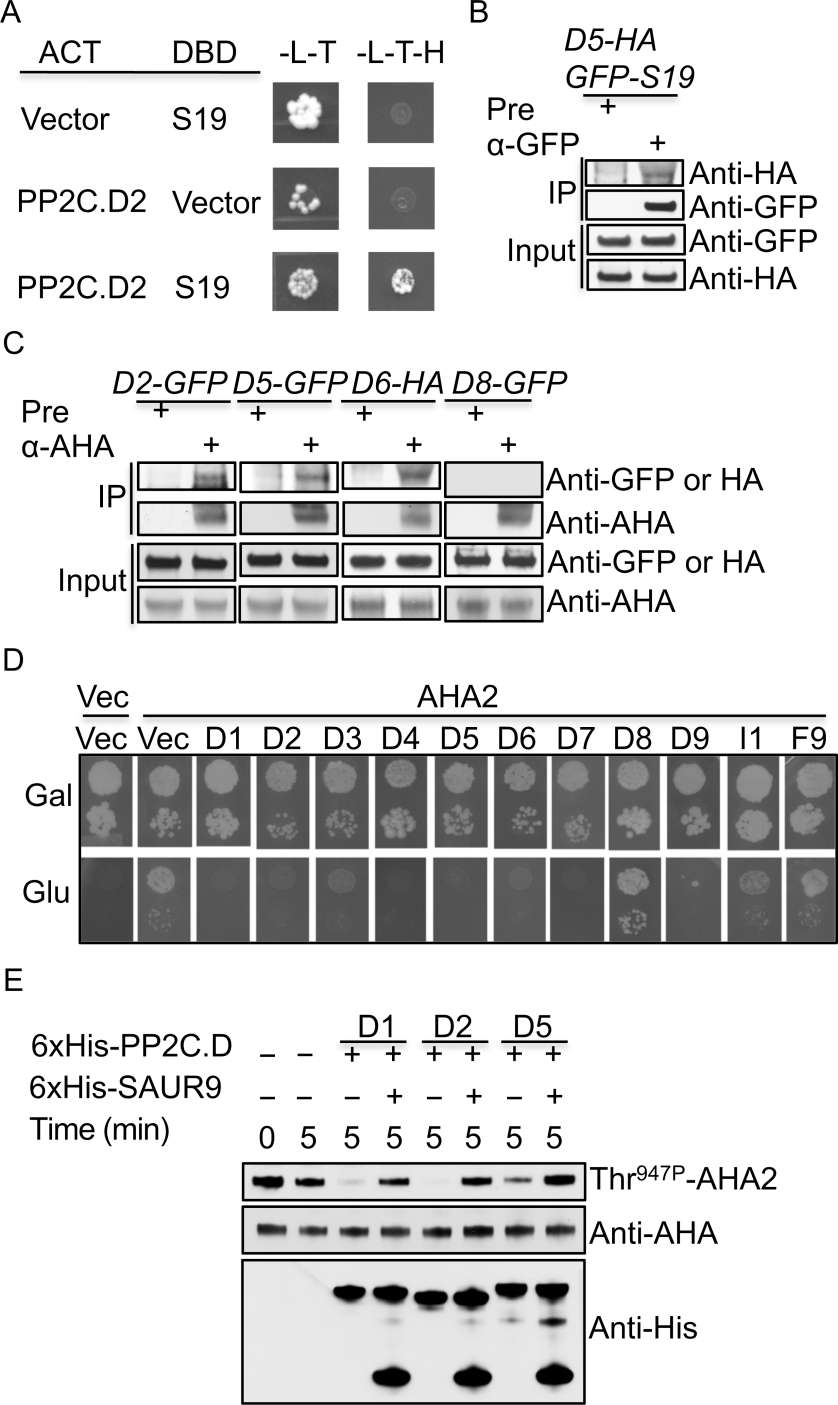
PP2C.D phosphatases interact with SAUR19 and plasma membrane H^+^-ATPases. **(**A) Yeast two-hybrid assay demonstrating PP2C.D2 and SAUR19 protein interaction. Cells were plated onto appropriate selection media and grown at room temperature for 3 to 6 days. (B) Co-IP assays detecting PP2C.D5-HA and GFP-SAUR19 protein interaction. Microsomal proteins were prepared from 6-day-old etiolated seedlings. Pre-immune bleed serum (pre) or anti-GFP antibody were used for the co-IP assays. PP2C.D5-HA and GFP-SAUR19 were detected by anti-HA and anti-GFP antibodies, respectively. (C) Co-IP assays detecting PP2C.D and PM H^+^-ATPase protein interactions. Microsomal proteins were prepared from 8-day-old light-grown seedlings. Pre-immune bleed serum (pre) or anti-AHA antibody were used for the co-IP assays. PP2C.D2-GFP, PP2C.D5-GFP, PP2C.D8-GFP, and PP2C.D6-HA proteins were detected by anti-GFP and anti-HA antibodies, respectively. (D) PP2C.D expression abolishes AHA2 complementation of PM H^+^-ATPase activity in yeast. Gal, galactose; Glu, glucose; Vec, empty vector. (E). *In vitro* AHA2 dephosphorylation assays examining the dephosphorylation of AHA2-Thr^947P^ expressed in yeast. (B and C) 300–400 μg and 10–20 μg of microsomal proteins were used for co-IP and western blots, respectively.

The above genetic studies revealed that PP2C.D2, D5, and D6 are the major D-clade phosphatases that negatively regulate SAUR-mediated cell expansion. Our previous work isolated PP2C.D1, D5, and D6 as SAUR19 interacting proteins in a yeast two-hybrid library screen [18]. We confirmed that PP2C.D2 also interacted with SAUR19 in this system (Fig 4A). We also tested the remaining PP2C.D family members and found that PP2C.D3, D4, and D8 can also interact with SAUR19 (S6A Fig). Positive interactions were not detected for PP2C.D7 or PP2C.D9 (S6A Fig), however, PP2C.D7 did not appear to be expressed in yeast and PP2C.D9 expression was quite low in comparison to PP2C.D1 (S6B Fig).

To examine protein interactions *in planta*, we generated transgenic Arabidopsis plants co-expressing PP2C.D2-HA, D5-HA, or D6-HA under the control of native *PP2C*.*D* promoters and GFP-SAUR19 driven by the *35S* promoter and examined their interactions by co-immunoprecipitation (co-IP) using solubilized microsomal fractions. PP2C.D5-HA and GFP-SAUR19 co-immunoprecipitated, confirming their interaction in Arabidopsis (Fig 4B). However, we could not successfully co-immunoprecipitate GFP-SAUR19 with PP2C.D2-HA and PP2C.D6-HA. While this may be due to the technical limitations of this assay, we cannot exclude the possibility that SAUR19 does not interact with these phosphatases *in planta*. Rather, given the large number of SAUR proteins, it seems quite possible that PP2C.D2 and D6 may preferentially interact with other plasma membrane-associated SAUR proteins.

The pronounced increase in AHA-Thr^947^ phosphorylation observed in the *pp2c*.*d2/5/6* triple mutant (Fig 3F) identified this phosphosite as a putative substrate of PP2C.D2, D5, and D6 phosphatases. We therefore examined potential interactions between these proteins and PM H^+^-ATPases by co-IP and bimolecular fluorescence complementation (BiFC) assays. PP2C.D2-GFP, D5-GFP, and D6-HA all co-immunoprecipitated with AHA proteins (Fig 4C). In contrast, no detectable interaction was observed between AHAs and PP2C.D8-GFP (Fig 4C), suggesting at least some degree of substrate specificity among the PP2C.D family members. Additionally, yellow fluorescent signals were observed at the plasma membrane of leaf epidermal cells when AHA2-YFP^N^ was transiently co-expressed with PP2C.D2-YFP^C^, D5-YFP^C^, and D6-YFP^C^ in *Nicotiana benthamiana* leaves (S7 Fig). These results indicate that PP2C.D2, D5, and D6 phosphatases physically associate with AHA proteins *in planta*. To further test the regulatory nature of these interactions, we co-expressed PP2C phosphatases with AHA2 in yeast strain RS-72 for complementation assays. In this strain, cells are only viable when grown on galactose media, since the endogenous yeast PM H^+^-ATPase gene *PMA1* is driven by the *GAL1* promoter. AHA2 expression complements *GAL1pro*:*PMA1* to restore growth on glucose media [33, 40]. When we co-expressed PP2C.D2, D5, or D6 with AHA2, yeast RS-72 cells were unable to grow on glucose media (Fig 4D), indicating that these phosphatases inhibit AHA2 function. In fact, all PP2C.D family members with the exception of PP2C.D8 were capable of antagonizing AHA2 function in yeast. In contrast, the non-D-clade Arabidopsis PP2C phosphatases PP2C.I1 (At2g25070, an I-clade PP2C) and PP2C.F9 (At1g43900, an F-clade PP2C) [41] failed to inhibit AHA2 function in this system (Fig 4D), suggesting that the D-clade PP2Cs may be unique in their ability to inhibit PM H^+^-ATPase activity. Presumably, PP2C.D1, D3, D4, and D9, all of which displayed some degree of cytosolic localization in Arabidopsis, can access the cytosolic C-terminus of AHA2 when overexpressed in yeast. Our genetic (S5 Fig) and biochemical (Fig 3F) findings, however, suggest that these family members are not the primary regulators of AHA activity *in planta*.

We previously developed an *in vitro* AHA2 dephosphorylation assay using plasma membranes prepared from yeast RS-72 cells expressing AHA2 to examine SAUR regulation of PP2C.D1-mediated dephosphorylation of AHA2-Thr^947P^ [18]. This same assay was used to assess the ability of PP2C.D2, D5, and D6 phosphatases to dephosphorylate AHA2-Thr^947P^ and the inhibition of any such activity by SAUR proteins. As previously shown for PP2C.D1 [18], recombinant PP2C.D2 and PP2C.D5 catalyzed AHA2-Thr^947^ dephosphorylation and this activity was strongly inhibited by the addition of purified SAUR9 protein (Fig 4E). We could not demonstrate phosphatase activity for recombinant PP2C.D6 in this system or in assays employing the chemical substrate *p*-nitrophenyl phosphate (pNPP), suggesting that this phosphatase may require alternative reaction conditions, co-factors, or post-translational modifications. That said, we cannot eliminate the possibility that PP2C.D6 is not a functional phosphatase, and rather may play a distinct role such as providing a scaffolding function for PP2C.D-substrate complexes. However, given that PP2C.D6 contains a highly conserved catalytic domain, together with our interaction data and genetic findings demonstrating that D6 functions redundantly with D2 and D5, it seems likely that PP2C.D6 also dephosphorylates AHA2-Thr^947P^ and this activity is inhibited by SAUR proteins.

### *PP2C*.*D5* overexpression confers reduced cell expansion and plant growth

To investigate the effects of *PP2C*.*D* gain-of-function on plant growth and development, we tried to generate *35Spro*:*PP2C*.*D5-EYFP* overexpression lines. While several primary Arabidopsis transformants exhibited a dwarf phenotype, we could not obtain any stable homozygous overexpression lines, suggesting that PP2C.D5 dosage may be critical. We therefore generated transgenic Arabidopsis plants expressing *PP2C*.*D5-HA* driven by the *PP2C*.*D5* promoter in the *pp2c*.*d5* mutant background. PP2C.D5-HA protein expression rescued the slightly increased hypocotyl growth phenotype of *pp2c*.*d5* seedlings (S8A Fig), demonstrating that PP2C.D5-HA is a functional protein. We noticed that some transgenic lines exhibited growth defects, including reduced growth and fertility. The severity of growth defects was dependent on the expression levels of PP2C.D5-HA protein. *pp2c*.*d5 PP2C*.*D5-HA* lines 6 and 7 that did not exhibit obvious growth defects expressed lower levels of PP2C.D5-HA protein, while lines 1 and 4 that exhibited severe growth defects expressed higher levels of PP2C.D5-HA protein (S8B Fig). We therefore selected *pp2c*.*d5 PP2C*.*D5-HA* lines 1 and 4 (hereafter referred to as *D5-HA-OX* for Over-eXpression) for further phenotypic analyses to assess the effects of *PP2C*.*D5* gain-of-function on plant growth and development.

**Fig 5:**
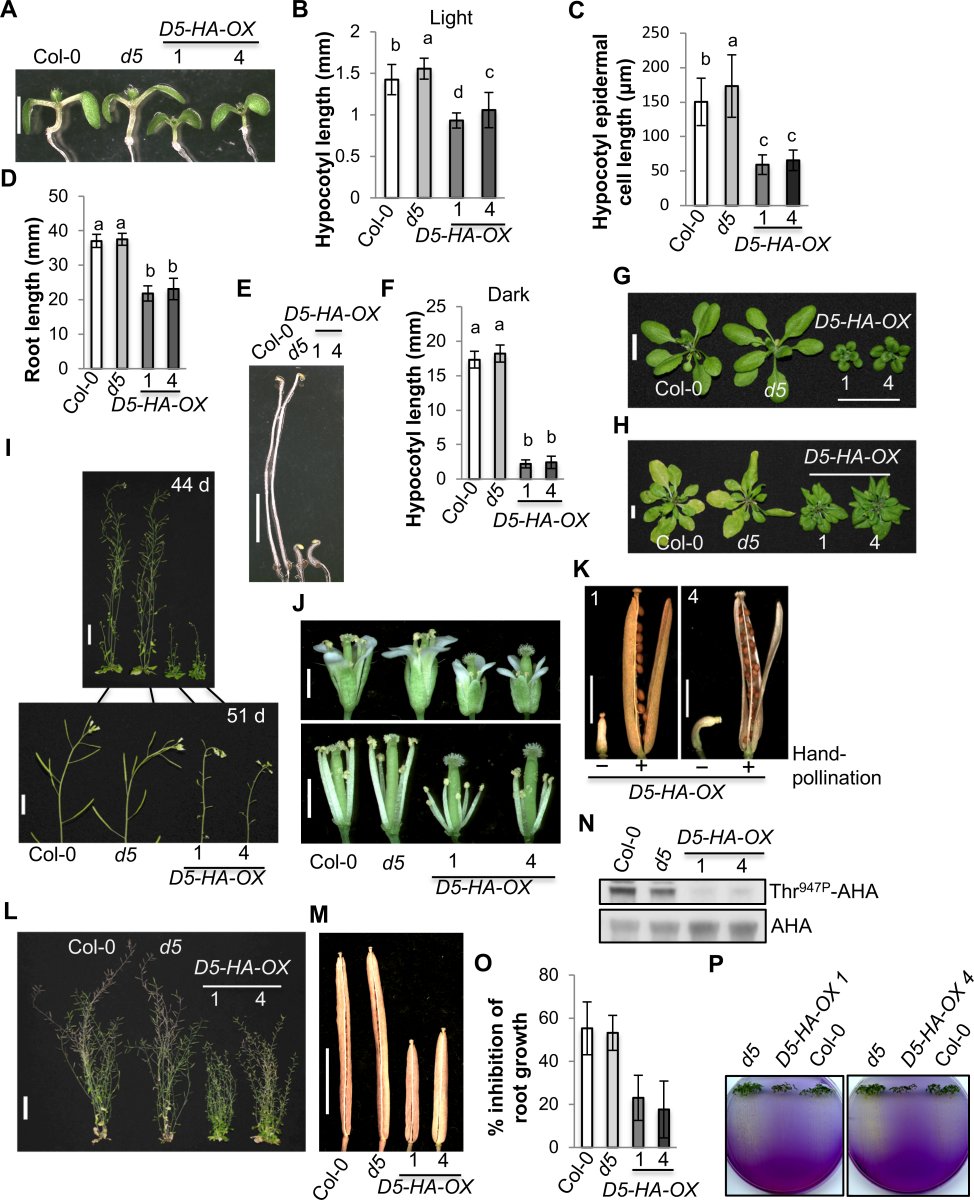
*PP2C*.*D5* overexpression confers reduced cell expansion and plant growth. **(**A) Shoots of 7-day-old light-grown seedlings. Scale bar = 2 mm. (B) Hypocotyl length of 7-day-old light-grown seedlings. Error bars = SD (n = 33–43). (C) Epidermal cell length of 8-day-old light-grown seedling hypocotyls. The apical most 10 cells from 10 seedlings were measured. Error bars = SD. (D) Primary root length of 7-day-old light-grown seedlings. Error bars = SD (n = 37–51). (E) Shoots of 7-day-old etiolated seedlings. Scale bar = 5 mm. (F) Hypocotyl length of 7-day-old etiolated seedlings. Error bars = SD (n = 40–49). (G) 24-day-old plants. Scale bar = 1 cm. (H) 44-day-old plants. Primary bolts were removed to better observe rosette leaves. Scale bar = 1 cm. (I) Shoots of 44 or 51-day-old plants. Scale bar = 4 cm (top) or 1 cm (bottom). (J) Flowers. Sepals and petals were removed to better observe stamen filaments. Scale bar = 1 mm. (K) Siliques. The pistils of *D5-HA-OX* lines 1 and 4 flowers were hand-pollinated with their own pollen grains. Scale bars = 2 mm. (L) 73-day-old plants. Scale bar = 4 cm. (M) Siliques. Scale bar = 4 mm. (N) Levels of Thr^947^-phosphorylated AHA proteins as monitored by GST-14-3-3 binding. Five micrograms of microsomal fractions prepared from 6-day-old etiolated seedlings were loaded. AHA and GST-14-3-3-bound AHA-Thr^947P^ proteins were detected by anti-AHA and anti-GST antibodies, respectively. (O) LiCl root inhibition assay. Six-day-old light-grown seedlings grown on ATS plates were transferred onto ATS or ATS + 10 mM LiCl plates for 3 days. New root growth after transfer was measured. Error bars = SD (n = 49–73). (P) Medium acidification assays. Eight-day-old light-grown seedlings grown on ATS plates were transferred to plates containing the pH indicator bromocresol purple (BCP, pH 6.5), and the medium color change was observed 9 (*D5-HA-OX* 1) or 12 (*D5-HA-OX* 4) days later. (B, C, D, and F) Different letters above the bars indicate significant differences (P < 0.05).

Compared with wild-type and *pp2c*.*d5* seedlings, light-grown *D5-HA-OX* seedlings exhibited reduced hypocotyl growth (Fig 5A and 5B), shorter hypocotyl epidermal cells (Fig 5C), and reduced root growth (Fig 5D). Etiolated *D5-HA-OX* seedlings also exhibited severely reduced hypocotyl growth (Fig 5E and 5F). *D5-HA-OX* plants exhibited smaller rosette leaves (Fig 5G), delayed leaf senescence (Fig 5H), and smaller flowers with shorter stamen filaments (Fig 5J). Shortly after bolting, *D5-HA-OX* plants exhibited fertility defects (Fig 5I). However, hand-pollination of *D5-HA-OX* pistils with *D5-HA-OX* pollen grains resulted in full seed set (Fig 5K), indicating that the fertility defects are caused by reduced stamen filament elongation rather than defective pollen or fertilization. Curiously, with continued growth, older *D5-HA-OX* plants recovered from the early male fertility defects and could set seeds successfully. The mature *D5-HA-OX* plants were smaller than those of wild-type and *pp2c*.*d5* (Fig 5L) and had shorter siliques (Fig 5M).

To assess whether *PP2C*.*D5* overexpression confers reduced PM H^+^-ATPase activity, we examined the phosphorylation status of AHA-Thr^947^ in *D5-HA-OX* seedlings. A clear decrease in AHA-Thr^947^ phosphorylation was observed in etiolated *D5-HA-OX* seedlings (Fig 5N). Consistent with a reduction in PM H^+^-ATPase activity and consequent membrane potential, *D5-HA-OX* seedlings exhibited resistance to 10 mM LiCl (Fig 5O) and reduced medium acidification (Fig 5P). Together, our results suggest that *PP2C*.*D5* overexpression confers reduced cell expansion and plant growth, which are caused at least in part by reduced PM H^+^-ATPase Thr^947^ phosphorylation and the corresponding reduction in enzyme activity.

### *GFP-SAUR19* overexpression suppresses the growth defects of *PP2C*.*D5* overexpression plants

**Fig 6:**
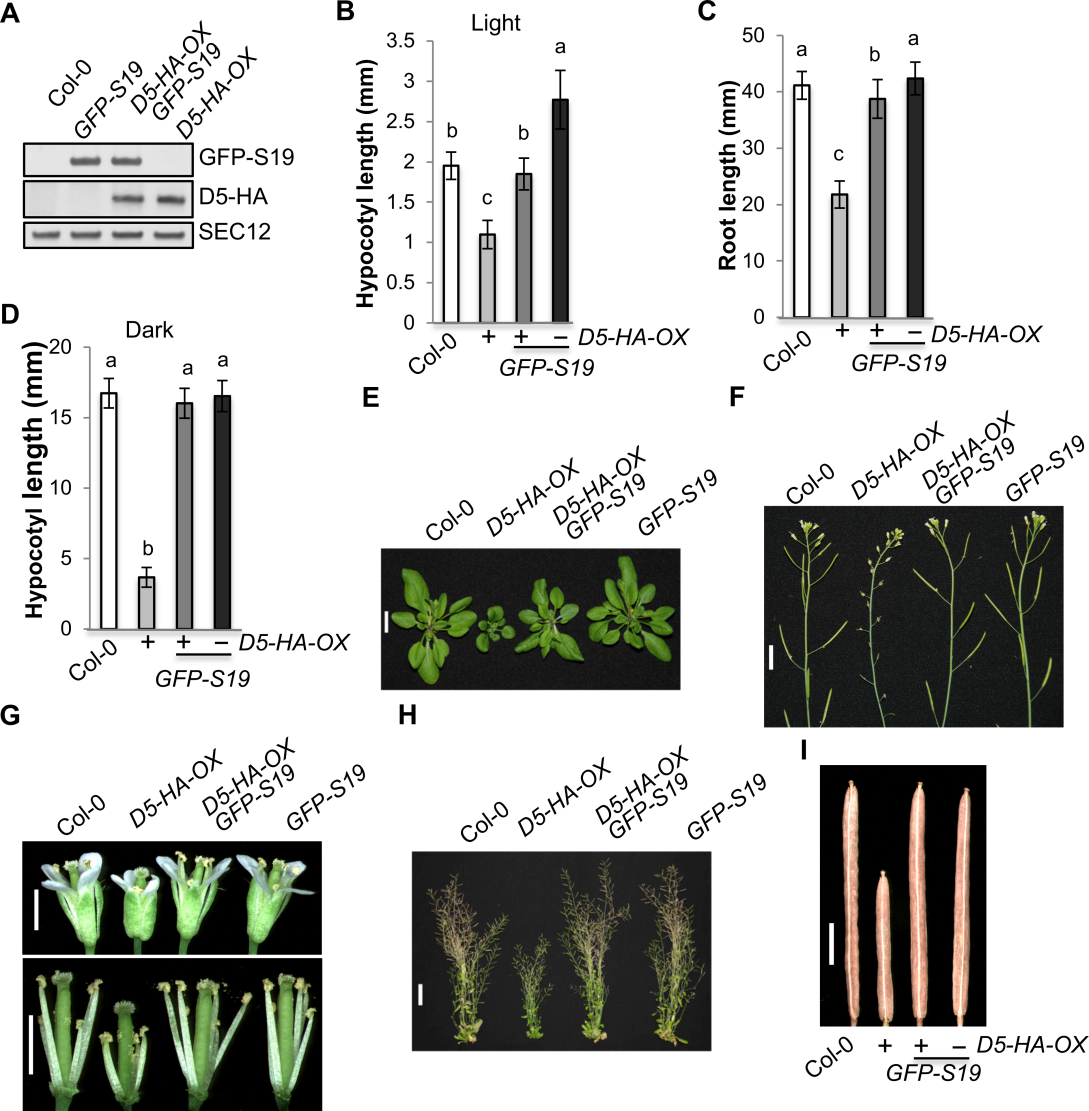
*GFP-SAUR19* overexpression suppresses the growth defects of *PP2C*.*D5* overexpression plants. (A) Western blot analyses of GFP-SAUR19 and PP2C.D5-HA protein expression. Twenty micrograms of microsomal proteins from 8-day-old light-grown plants were loaded. GFP-SAUR19, PP2C.D5-HA, and the SEC12 loading control were detected by anti-GFP, anti-HA, and anti-SEC12 antibodies, respectively. (B) Hypocotyl length of 8-day-old light-grown seedlings. Error bars = SD (n = 33–53). (C) Primary root length of 8-day-old light-grown seedlings. Error bars = SD (n = 41–51). (D) Hypocotyl length of 7-day-old etiolated seedlings. Error bars = SD (n = 68–78). (E) 30-day-old plants. Primary bolts were removed to better observe rosette leaves. Scale bar = 1 cm. (F) Apex of 53-day-old plants. Scale bar = 1 cm. (G) Flowers. Scale bars = 2 mm. Sepals and petals were removed to better observe stamen filaments. (H) 66-day-old plants. Scale bar = 4 cm. (I) Siliques. Scale bar = 2 mm. (B-D) Different letters above the bars indicate significant differences (P < 0.05).

The Arabidopsis genome contains 79 *SAUR* genes. Due to extensive functional redundancy, it is challenging to study the functions of SAUR proteins in plant growth and development using a loss-of-function approach [14]. Our prior biochemical studies have demonstrated that SAUR proteins inhibit PP2C.D phosphatase activity (Fig 4E) [18]. To test this hypothesis genetically, we generated Arabidopsis plants co-expressing GFP-SAUR19 and PP2C.D5-HA proteins by crossing the *35Spro*:*GFP-SAUR19* transgene into *D5-HA-OX* line 1, and examined the effects of *GFP-SAUR19* overexpression on the growth defects of *PP2C*.*D5-HA* overexpression plants. Western blot analysis confirmed that the double transgenic plants expressed both fusion proteins at levels comparable to that seen in the parental lines (Fig 6A). Strikingly, *GFP-SAUR19* overexpression suppressed virtually all aspects of the *D5-HA-OX* phenotypes, including the hypocotyl, root, and rosette leaf growth defects (Fig 6B–6E). In addition, the male sterility of *D5-HA-OX* plants caused by defective stamen filament elongation growth was also suppressed by *GFP-SAUR19* overexpression (Fig 6F and 6G), as were the D5-HA-OX defects in plant height and silique length (Fig 6H and 6I). Together, these results provide strong genetic support for the hypothesis that SAUR proteins and plasma membrane-localized PP2C.D phosphatases function antagonistically to regulate plant growth.

### Auxin induces the expression of a subset of *PP2C*.*D* genes and high temperature upregulates PP2C.D2 protein levels

**Fig 7:**
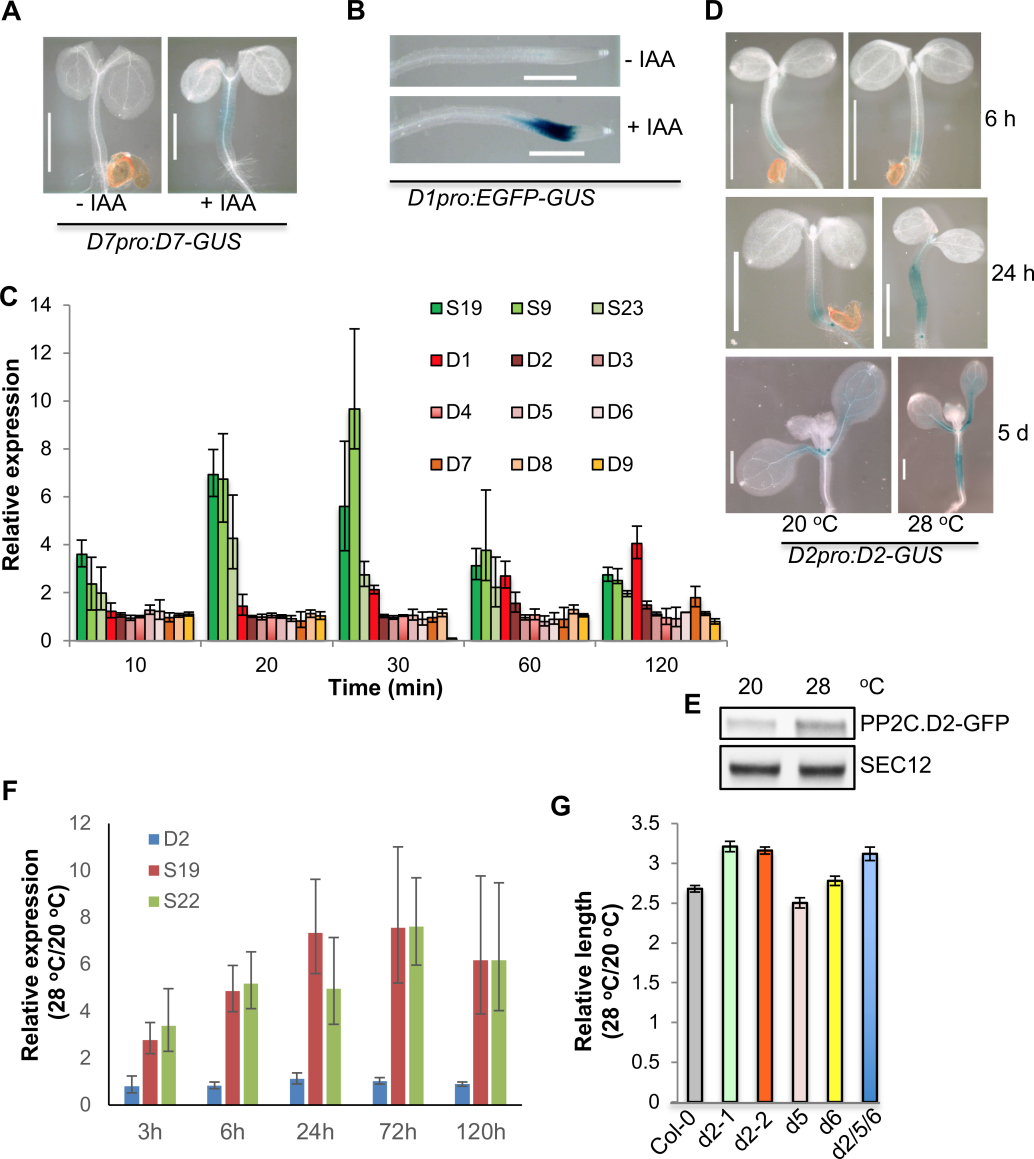
Auxin induces *PP2C*.*D* gene expression, and high temperature upregulates PP2C.D2 protein levels. (A) GUS-stained shoots of 5-day-old light-grown seedlings treated with 10 μM IAA for 4 h. Scale bars = 1 mm. (B) GUS-stained roots of 5-day-old light-grown seedlings treated with 10 μM IAA for 4 h. Scale bars = 1 mm. (C) qRT-PCR analyses of *SAUR* and *PP2C*.*D* gene expression. RNA was prepared from 3-day-old light-grown seedlings treated with 5 μM NAA or solvent control for various times. Relative expression represents expression value of NAA/expression value of mock. qRT-PCR results were based on three biological replicates. *S19*, *SAUR19*; *S23*, *SAUR23*; *S9*, *SAUR9*. Error bars = SD. (D) GUS-stained shoots of light-grown seedlings. Two or four-day-old *PP2C*.*D2-GUS* seedlings grown at 20 ^o^C were shifted to 28 ^o^C for 6 h, 24 h, or 5 d. GUS staining was performed at 37 ^o^C for 2 h (6 h, 24 h) or 1 h (5 d). (E) Western blot analysis of PP2C.D2-GFP protein expression. Two-day-old *PP2C*.*D2-GFP* seedlings grown at 20 ^o^C were shifted to 28 ^o^C for 5 days. 25 micrograms of total proteins from shoots were loaded for western blot analyses using anti-GFP and anti-SEC12 antibodies. (F) qRT-PCR analyses of *SAUR* and *PP2C*.*D* gene expression. RNA was prepared from light-grown seedlings that were grown at 20 ^o^C and shifted to 28 ^o^C for various times. Relative expression represents expression value of 28 ^o^C/expression value of 20 ^o^C. qRT-PCR results were based on three biological replicates. Error bars = SD. (G) Relative hypocotyl length of 7-day-old light-grown seedlings. Relative hypocotyl length represents length value of 28 ^o^C/length value of 20 ^o^C. Error bars = SEM (n ≥ 22).

Previous hypocotyl transcriptomic analyses of auxin-responsive genes revealed that *PP2C*.*D1* and *PP2C*.*D7* may be auxin-induced genes, as their expression was upregulated by a 120 min treatment with the synthetic auxin picloram [42]. Additionally, using our PP2C.D1-GFP reporter, we demonstrated auxin-inducible expression of *PP2C*.*D1* in root tips (Fig 2A). To examine potential auxin-mediated regulation of *PP2C*.*D* family genes, we examined *PP2C*.*D-GUS* expression in 5-day-old light-grown *PP2C*.*D1pro*:*EGFP-GUS* and *PP2C*.*D(2–9)pro*:*PP2C*.*D(2–9)-GUS* seedlings treated with 10 μM IAA. Consistent with previous findings [42], auxin induced *PP2C*.*D7-GUS* expression in the hypocotyls (Fig 7A). Under our conditions, auxin induction of *PP2C*.*D1pro*:*EGFP-GUS* expression was not apparent in the hypocotyls. However, auxin strongly induced *PP2C*.*D1pro*:*EGFP-GUS* expression in the root elongation zone (Fig 7B). We did not observe obvious auxin-induced expression of the PP2C.D2-, D3-, D4-, D5-, D6-, D8-, or D9-GUS reporters.

To more precisely study the kinetics of auxin-induction of *SAUR* and *PP2C*.*D* gene expression, we examined the transcript levels of *SAUR9*, *19*, *23*, and *PP2C*.*D1-9* genes in 3-day-old light-grown wild-type seedlings treated with 5 μM NAA over a 2 h time-course by qRT-PCR. Auxin-induction of *SAUR* gene expression was observed within 10 minutes, and the induction peaked at 20–30 minutes (Fig 7C). While auxin also induced *PP2C*.*D1* and *PP2C*.*D7* gene expression, the kinetics were noticeably delayed in comparison to the *SAUR* genes examined. *PP2C*.*D1* was slightly up-regulated at 30 minutes and expression continued to increase throughout the 2 h time course, while *PP2C*.*D7* expression was not elevated until 2 hours of auxin treatment (Fig 7C). These results indicate that compared with the *SAUR* genes, *PP2C*.*D1* and *PP2C*.*D7* exhibit a delayed transcriptional response to auxin treatments.

High temperature induces the expression of the *SAUR19* family genes to promote hypocotyl growth [43]. We were curious whether high temperature might also affect *PP2C*.*D* gene expression. *PP2C*.*D1pro*:*EGFP-GUS* and *PP2C*.*D(2–9)pro*:*PP2C*.*D(2–9)-GUS* seedlings were shifted from 20 ^o^C to 28 ^o^C for 24 hours or 5 days and then stained alongside control seedlings maintained at 20 ^o^C. Only *PP2C*.*D2pro*:*PP2C*.*D2-GUS* seedlings exhibited increased GUS staining in the hypocotyls (Figs 7D and S9). We therefore conducted a time-course to more carefully examine PP2C.D2 expression in *PP2C*.*D2pro*:*PP2C*.*D2-GUS* seedlings shifted from 20 ^o^C to 28 ^o^C. While no difference was observed at early time points (6 h), strong GUS staining in the hypocotyls and petioles of seedlings shifted to 28 ^o^C was seen at the later time points (Fig 7D). To gain additional evidence to support that high temperature upregulates PP2C.D2 protein levels, we examined PP2C.D2-GFP protein levels in *PP2C*.*D2pro*:*PP2C*.*D2-GFP* seedlings shifted from 20 ^o^C to 28 ^o^C. Increased PP2C.D2-GFP protein levels were detected in the shoots of seedlings shifted to 28 ^o^C for 5 days (Fig 7E). Together, these results indicate that high temperature upregulates PP2C.D2 protein levels. Since both the GUS and GFP reporters are translational fusions, this temperature-dependent increase could be the result of transcriptional or post-transcriptional regulation. To determine if this effect was the result of increased transcription, we examined *PP2C*.*D2* transcript levels in light-grown wild-type seedlings shifted to 28 ^o^C over a 120 h time-course by qRT-PCR. As previously reported [43], high temperature induced the expression of *SAUR19* and *SAUR22* within 3 hours, and transcript levels remained elevated throughout the time-course (Fig 7F). However, high temperature did not result in elevated *PP2C*.*D2* transcript levels, suggesting that the observed temperature-dependent increases in PP2C.D2-GUS and PP2C.D2-GFP protein levels must occur post-transcriptionally. These results suggest that unlike the *SAUR19* family genes, which display a rapid transcriptional response to high temperature, PP2C.D2 exhibits a delayed, post-transcriptional increase in protein abundance.

Antagonistic regulation of cell expansion by SAUR proteins and the PP2C.D2 phosphatase led us to hypothesize that high temperature upregulation of PP2C.D2 protein abundance may represent an additional layer of control to prevent cell overexpansion conferred by increased *SAUR* gene expression, which may cause plant overgrowth. To test this hypothesis, we examined the hypocotyl growth responses of *pp2c*.*d2-1* and *pp2c*.*d2-2* loss-of-function mutants to high temperature. Two-day-old wild-type seedlings grown at 20 ^o^C were shifted to 28 ^o^C for 5 days, and hypocotyl growth was assessed. Indeed, both *pp2c*.*d2* mutant lines exhibited an enhanced response to high temperature compared to wild-type controls (Fig 7G). In contrast, the *pp2c*.*d5* and *pp2c*.*d6* single mutants exhibited temperature-dependent increases in hypocotyl length that were comparable to wild-type. Furthermore, while the *pp2c*.*d2/5/6* triple mutant also exhibited enhanced elongation at high temperature, this increase was no more severe than that observed with the *pp2c*.*d2* single mutants. These findings suggest that high temperature specifically upregulates PP2C.D2 protein abundance to prevent hypocotyl overgrowth.

## Discussion

Cell expansion is a fundamental cellular process that is essential for plant growth and development. Our prior work suggested that plasma membrane-localized SAUR proteins inhibit PP2C.D phosphatase activity to activate PM H^+^-ATPases, thereby promoting cell expansion [18]. Using the Arabidopsis hypocotyl system as a model, we demonstrate that the exclusively plasma membrane-localized PP2C.D2, D5, and D6 phosphatases negatively regulate hypocotyl growth (Figs 2A and S5A). These proteins physically associate with PM H^+^-ATPases and negatively control cell expansion by dephosphorylating the penultimate threonine residue of PM H^+^-ATPases (Fig 4). Furthermore, we demonstrate that all three phosphatases can interact with SAUR19 in yeast 2-hybrid assays, that D5 co-immunoprecipitates with SAUR19 from plant extracts, and that the enzymatic activities of PP2C.D2 and D5 are inhibited by SAUR binding. While the *pp2c*.*d2/5/6* triple mutant largely phenocopies *GFP-SAUR19* overexpression plants, it is noteworthy that the hypocotyls of light-grown *pp2c*.*d2/5/6* seedlings were still slightly shorter than those of *GFP-SAUR19* seedlings (Fig 3A and 3B). This suggests that in addition to the PP2C.D2, D5, and D6 phosphatases, additional PP2C.D family members may make minor contributions to the control of hypocotyl length. Consistent with this possibility, all family members except *PP2C*.*D7* were strongly expressed in hypocotyls (Fig 1A), and we found that all except *PP2C*.*D8* could antagonize AHA2 function in the heterologous yeast expression system (Fig 4D). Alternatively, it is also possible that SAUR19 has regulatory targets in addition to the PP2C.D phosphatases that may contribute to SAUR19-mediated cell expansion. Other than PP2C.D2, D5, and D6, the remaining PP2C.D family members did not appear to influence cell expansion in hypocotyl growth (S5A Fig). However, these proteins may regulate cell expansion to control the growth of other specific organs or developmental processes. Supporting this hypothesis, PP2C.D1 was found to regulate the differential growth at the apical hook in etiolated seedlings [18, 24]. Our findings elucidate the contributions of individual PP2C.D phosphatases to cell expansion and provide insights into auxin-induced cell expansion via an acid growth mechanism.

Prior computational analysis of PP2C.D proteins identified putative bipartite nuclear localization signals in all 9 family members and potential transmembrane spanning regions in PP2C.D1, D3, D4, D6, D7, and D9 [41]. Our analysis of *PP2C*.*D(1–9)pro*:*PP2C*.*D(1–9)-GFP* reporters, as well as a prior study employing *35Spro*:*PP2C*.*D-GFP* reporters [28], correlate relatively poorly with these predictions. While PP2C.D2, D5, and D6 localized exclusively to the plasma membrane, the remaining PP2C.D family members localized to various cellular compartments, with D8 exhibiting mitochondrial localization, and D1, D3, and D4 exhibiting both nuclear and cytosolic localization (Fig 2). In the case of PP2C.D2, D5, and D6, two of which lack a predicted transmembrane span, it is unclear how they associate with the plasma membrane. However, since all three physically interact with PM H^+^-ATPases (Figs 4C and S7), an attractive hypothesis is that they are recruited to the plasma membrane via their interaction with these H^+^ pumps. In regard to the non-plasma membrane family members, it is interesting to note that some SAUR proteins have also been shown to reside in the nucleus (SAUR32 [44] and SAUR36 [45]) and cytosol (SAUR32 [44], SAUR40 [46], SAUR41 [47], SAUR55 [45], and SAUR71 [46]). However, the functions of SAUR proteins in these cellular compartments remain to be elucidated. We hypothesize that these SAUR proteins may regulate similarly localized PP2C.D proteins to control the phosphorylation status of their respective substrates. Identifying non-plasma membrane localized SAUR-PP2C.D regulatory modules and their substrates is an exciting area for future studies.

Our loss- and gain-of-function studies demonstrate that the PP2C.D2, D5, and D6 phosphatases function as negative regulators to control diverse plant growth processes, including root, hypocotyl, leaf, flower, and silique growth. These proteins play a crucial role in elongation growth, such as hypocotyl and stamen filament growth (Figs 3 and 5). Stamen filament elongation growth during the late stages of stamen development is crucial for mature pollen to reach the stigma for a successful pollination. The *yuc1/2/6*, *tir1 afb1/2/3*, and *arf6 arf8* mutants exhibit short stamen filaments [48–50], indicating an essential function of auxin in stamen filament elongation growth. The auxin-induced *SAUR63* family genes (*SAUR61-68* and *SAUR75*), the likely downstream targets of auxin response factors ARF6 and ARF8 [50], have been shown to positively regulate stamen filament elongation growth [34], suggesting that these SAUR proteins may contribute to auxin-mediated stamen filament elongation growth. The *PP2C*.*D2*, *D5*, and *D6* genes were all highly expressed in stamen filaments (S1B Fig), and *pp2c*.*d2/5/6* and *PP2C*.*D5* overexpression flowers exhibited longer and shorter stamen filaments, respectively, than those of wild-type flowers (Figs 3I and 5J). These results convincingly show that the PP2C.D2, D5, and D6 phosphatases negatively regulate stamen filament elongation growth and may therefore be important for the reproductive success of plants. It would be interesting to determine whether these three phosphatases physically interact with the SAUR63 family proteins to regulate stamen filament elongation growth. Our detailed phenotypic analyses of *pp2c*.*d2/5/6* and *GFP-SAUR19* plants indicate that *pp2c*.*d2/5/6* plants phenocopy nearly all known phenotypes of *GFP-SAUR19* overexpression plants, including increased cell expansion, hypocotyl and leaf growth, defective apical hook development and phototropic response, and elevated PM H^+^-ATPase phosphorylation and activity (Fig 3). These findings suggest that PP2C.D2, D5, and D6 phosphatases are the primary effectors of plasma membrane-localized SAUR proteins that regulate cell expansion.

Differential growth is crucial for plant development and growth responses to environmental stimuli, such as light and gravity. Our studies implicate PP2C.D2, D5, and D6 as important regulators of differential growth, as the triple mutant exhibits defects in both phototropic bending and apical hook development. We previously reported that while the *pp2c*.*d1* mutant exhibits defects in apical hook development, the *pp2c*.*d2*, *d5*, and *d6* single mutants displayed apical hooks comparable to wild-type controls [18]. However, consistent with our hypothesis that these phosphatases function redundantly, etiolated *pp2c*.*d2/5/6* seedlings exhibited clear defects in apical hook formation (Fig 3J and 3K). Interestingly, while *PP2C*.*D1* is specifically expressed on the inner side of apical hooks (Figs 1C, 1D and 2B), *PP2C*.*D2*, *D5*, and *D6* expression appears uniform across the hook (Fig 1C). Together with the observed differences in subcellular localization (Fig 2), these findings suggest that PP2C.D1 and the three PM-localized phosphatases likely play distinct roles in modulating apical hook development. Furthermore, unlike *PP2C*.*D1*, the uniform expression of the PM-localized family members across the apical hook suggests that regulatory proteins must be differentially expressed in order to achieve PP2C.D2/D5/D6-mediated differential growth. As auxin plays a prominent role in hook development, and auxin response is known to vary across the apical hook [51], it seems likely that auxin-regulated SAUR proteins fulfill this function. Support for this contention can be found from prior gene expression studies of tropically-stimulated organs, which have revealed that multiple *SAUR* genes are differentially expressed in tropically-stimulated stems and hypocotyls, with expression being higher on the elongating side of the organ [37–39, 52–55]. Since SAUR proteins inhibit PP2C.D activity, this differential pattern of *SAUR* expression would be expected to result in differential PP2C.D (and consequently PM H^+^-ATPase) activities across the organ, thus resulting in tropic bending. Indeed, differential apoplastic acidification was recently reported in gravistimulated Arabidopsis hypocotyls [20]. In the *pp2c*.*d2/5/6* mutant, however, the primary effectors of SAUR function are missing, and consequently, defects in apical hook development and tropic bending result.

While *SAUR* genes are rapidly upregulated in response to auxin, we did not observe auxin-mediated regulation of the majority of *PP2C*.*D* family members in either qRT-PCR or GUS reporter assays (Fig 7C). *PP2C*.*D1* and *PP2C*.*D7* were notable exceptions, however, as both genes were auxin-inducible, albeit with delayed kinetics compared to *SAUR* genes (Fig 7C). Although neither PP2C.D1 nor PP2C.D7 appear to play an important role in hypocotyl elongation under standard growth conditions, it is possible that delayed, auxin-induced expression could potentially function to attenuate SAUR-mediated growth regulation of other organs or processes, such as apical hook development.

A more compelling case for increased PP2C.D expression functioning to attenuate SAUR-mediated growth was observed in our studies of high temperature-induced hypocotyl elongation. Arabidopsis seedlings grown under high temperature conditions exhibit elongated hypocotyls [56, 57]. Our previous studies demonstrated that high temperature induces the expression of *SAUR19* family genes to promote hypocotyl growth [43]. In this study, we found that high temperature also specifically upregulates PP2C.D2 protein levels (Figs 7D, 7E and S9). This increase appears to occur post-transcriptionally, as temperature did not affect *PP2C*.*D2* mRNA levels. Compared with high temperature-induction of *SAUR* genes, the increase in PP2C.D2 protein abundance exhibited a delayed response to high temperature (Fig 7D and 7F). We demonstrate that this increase in PP2C.D2 expression is biologically meaningful, as two independent *pp2c*.*d2* mutant seedlings exhibited enhanced hypocotyl growth under high temperature conditions (Fig 7G). These results provide strong support for the hypothesis that high temperature upregulation of PP2C.D2 protein abundance attenuates the hypocotyl growth conferred by SAUR proteins, preventing hypocotyl overgrowth. Our findings suggest a novel layer of regulation in high temperature-induced elongation growth. Determining the underlying mechanism(s) by which temperature affects PP2C.D2 protein abundance, such as enhanced translation or increased protein stability, is an exciting topic for future research that will further elucidate our understanding of high temperature-induced growth regulation.

## Materials and methods

### Plant materials and growth conditions

Arabidopsis Genome Initiative locus identifiers for the genes employed in this study are as follows: *SAUR19* (At5g18010), *SAUR9* (At4g36110), SAUR22 (At5g18050), SAUR23 (At5g18060), *PP2C*.*D1* (At5g02760), *PP2C*.*D2* (At3g17090), *PP2C*.*D3* (At3g12620), *PP2C*.*D4* (At3g55050), *PP2C*.*D5* (At4g38520), *PP2C*.*D6* (At3g51370), *PP2C*.*D7* (At5g66080), *PP2C*.*D8* (At4g33920), *PP2C*.*D9* (At5g06750), *PP2C*.*I1* (At2g25070), *PP2C*.*F9* (At1g43900), *AHA2* (4g31090), and *AUX1* (At2g38120).

*Arabidopsis thaliana* plants were grown under long-day conditions (16 h light/8 h dark) under ~ 80 μEm^-2^s^-1^ fluorescent lighting at 20–22 ^o^C unless stated otherwise in figure legends. All transgenic lines and mutants were in the Columbia (Col) ecotype. Seeds were surface sterilized in a solution containing 30% bleach and 0.04% triton X-100 and washed in sterile water. Seeds were cold treated for 2 to 3 days at 4 ^o^C to synchronize germination. Seedlings were grown on ATS plates containing 1% sucrose and 0.5% Agargel (Sigma-Aldrich). The ATS nutrient solution contained 5 mM KNO_3_, 2.5 mM KPO_4_, 2 mM MgSO_4_, 2 mM Ca(NO_3_)_2_, 50 μM Fe-EDTA, 70 μM H_3_BO_3_,14 μM MnCI_2_, 0.5 μM CuSO_4_, 1 μM ZnSO_4_, 0.2 μM Na_2_MoO_4_,10 μM NaCI, and 0.01 μM CoCI_2_. For medium acidification assays, 6–8 day-old light-grown seedlings were transferred from ATS medium to plates containing 0.04 mg/ml bromocresol purple (BCP, Sigma) and 0.5% Agargel with the pH adjusted to 6.5 with KOH, and incubated under long-day lighting as detailed above. Once color changes to the medium were visible, plates were imaged on a bed scanner.

### Statistical analyses

All statistical analyses were performed by analysis of variance (ANOVA) with the JMP Pro 13.1 software suite (SAS Institute). Results of Tukey’s HSD (honestly significant difference) test were grouped by letters, with different letters indicating significant differences (P < 0.05).

### Phototropism analyses

Sterilized seeds were cold treated for 3 days at 4°C in the dark, exposed to white light for 2 hours at 20 ^o^C to induce seed germination, and then incubated for 4 days at 20 ^o^C in the dark. Etiolated seedlings were photo-stimulated with unilateral blue light for various times, and the angles of hypocotyl bending were measured by ImageJ. Blue light (470 nm) was provided by a SNAP LITE light system (Quantum Devices).

### Transgenic plant lines

*PP2C*.*D1-D9* genomic DNAs containing the promoters and coding sequences without the stop codons were amplified by PCR (S1 Table) and cloned into pENTR/D-TOPO using the pENTR directional TOPO cloning kit (Invitrogen). In most cases, the entire intergenic region between the *PP2C*.*D* start codon and the previous gene was included. The length of upstream promoter sequence for each gene is listed in S1 Table. These *PP2C*.*D* inserts were recombined into pGWB203 (GUS) [58], pGWB204 (GFP) [58], and pEarleyGate 301 (HA) [59] using the Gateway LR clonase II enzyme mix (Invitrogen) to make *PP2C*.*D(2–9)pro*:*PP2C*.*D(2–9)-GUS*, *PP2C*.*D(1–9)pro*:*PP2C*.*D(1–9)-GFP*, and *PP2C*.*D(2*,*5*,*6)pro*:*PP2C*.*D(2*,*5*,*6)-HA* constructs, respectively. Similarly, 4426 bp of *PP2C*.*D1* promoter sequence was cloned into pBGWFS7 (EGFP-GUS) [60] to make a *PP2C*.*D1pro*:*EGFP-GUS* construct. All binary vectors were introduced into *Agrobacterium tumefaciens* strain GV3101 (helper plasmid pMP90) by electroporation. The floral dip method was used to transform Arabidopsis [61]. Transgenic plants were selected on ATS plates containing 0.01% herbicide Basta (Bayer CropScience) or hygromycin (25 μg/ml).

### GUS assays

GUS staining of plant tissues was performed at 37 ^o^C in a solution containing 100 mM sodium phosphate (pH 7.0), 10 mM EDTA, 0.5 mM K_4_Fe[CN]_6_, 0.5 mM K_3_Fe[CN]_6_, 0.1% triton X-100, and 1 mM X-Gluc (5-Bromo-4-chloro-3-indoxyl-beta-D-glucuronide cyclohexylammonium salt, Gold Biotechnology). After removing chlorophyll with 70% ethanol, GUS-stained tissues were cleared in a solution containing 20% lactic acid and 20% glycerol. GUS expression patterns were imaged with an Olympus SZX12 dissecting microscope using the SPOT Advanced imaging software.

### Co-immunoprecipitation (co-IP) and western/far-western blots

Plant microsomal proteins were prepared as previously described [62]. Co-IP and western blot assays were performed as previously described with the exception that the tris-buffered saline buffer (TBST, 0.05% tween 20, pH7.6) was used for western blots [18]. Far-western blot assays were performed as previously described [32]. GST-14-3-3 fusion proteins were detected by an HRP-conjugated anti-GST antibody (GE Healthcare Life Sciences). Proteins were detected using the SuperSignal West Pico or West Femto Maximum Sensitivity Substrates (Thermo Scientific).

### Yeast two-hybrid and complementation assays

The lexA-based yeast two-hybrid system using the bait plasmid pBTM116 and the prey plasmid pACT2 [63] was used to examine SAUR19 and PP2C.D phosphatase interactions. pBTM116-SAUR19 and pACT2-PP2C.D plasmids were co-transformed into yeast strain L40ccU3 [*MATa*, *his3-200*, *trp1-901*, *leu2-3*, *112ade2 LYS2*::*(lexAop)4-HIS3*, *URA*::*(lexAop)8-lacZ*, *GAL4*, *gal80*] [63], plated onto appropriate selection media, and grown at room temperature for 3 to 6 days.

*Saccharomyces cerevisiae* strain RS-72 (*MATa*, *ade1-100*, *his4-519*, *leu2-3*, *312*, *GAL1pro*:*PMA1*) and the *PMA1pro*:*AHA2* construct in the expression plasmid pMP1745 were previously described [40]. *PP2C*.*D1-9*, *PP2C*.*I1*, and *PP2C*.*F9* full-length cDNAs were cloned into the *Not*I site of the expression vector pMP1612 [40]. All plasmids were introduced into yeast strain RS-72 by lithium acetate transformation. *PMA1* complementation tests were performed as previously described [18].

### *In vitro* AHA2 dephosphorylation assays

The *6xHis-SAUR9* and *6xHis-PP2C*.*D1* constructs in the expression vector pET32 were previously described [18]. The full-length cDNAs of *PP2C*.*D2* and *PP2C*.*D5* in pENTR/D-TOPO were recombined into pET32-GW using the Gateway LR clonase II enzyme mix to make the *6xHis-PP2C*.*D2* and *6xHis-PP2C*.*D5* bacterial expression constructs. Expression and purification of His-tagged recombinant proteins and AHA2 dephosphorylation assays were carried out as previously described [18].

### Quantitative RT-PCR

RNAs were prepared from seedlings using the RNeasy Plant Mini (Qiagen) or Nucleospin RNA Plant (Macherey-Nagel) kit, and an on-column DNase treatment was included to remove contaminating DNA. Two micrograms of RNA were used to synthesize cDNA using Moloney murine leukemia virus (M-MLV) reverse transcriptase (Promega). qRT-PCR reactions were performed on the LightCycler System (Roche Applied Sciences) using the SYBR Green JumpStart Taq Ready Mix (Sigma-Aldrich) or StepOnePlus Real-Time PCR System (Applied Biosystems) using the Brilliant III Ultra-Fast SYBR Green QPCR Master Mix (Agilent Genomics). Primers for qRT-PCR were previously described [18, 36], and results were based on three biological replicates.

### Bimolecular fluorescence complementation (BiFC) assays

The full-length cDNA sequences lacking stop codons of *PP2C*.*D2*, *D5*, and *D6* were cloned into pENTR/D-TOPO. These inserts were subsequently recombined into the pSPYNE and pSPYCE destination vectors [64] using Gateway LR Clonase II Enzyme Mix to generate BiFC expression constructs. *AHA2* and *AUX1* BiFC expression constructs were previously described [18]. All binary vectors were introduced into Agrobacterium strain GV3101 (with the pMP90 helper plasmid) by electroporation. BiFC assays were performed in an *Nicotiana benthamiana* transient expression system as previously described [65]. Leaves of ~ 5-week-old *Nicotiana benthamiana* plants were used for infiltration. The infiltration solution contained 10 mM MgCl_2_, 10 mM MES-KOH (pH 5.6), and 150 μM acetosyringone. Fluorescent signals in leaf epidermal cells were observed three days after infiltration. Confocal microscopy was performed with a Nikon A1 spectral confocal microscope.

## Supporting information

S1 Fig Expression patterns of *PP2C*.*D-GUS* reporters. β-glucuronidase staining patterns of (A) 2-week-old light-grown plants, (B) flowers, and (C) siliques. GUS staining was performed at 37 ^o^C for 24 h. Scale bars = 2 mm (A and C) or 1 mm (B).
(PDF)

S2 Fig Expression patterns of *PP2C*.*D7*. This figure was obtained from the Arabidopsis eFP Browser (http://bar.utoronto.ca/efp/cgi-bin/efpWeb.cgi).
(PDF)

S3 Fig *pp2c*.*d* T-DNA insertion mutants. (A) T-DNA insertion locations in *PP2C*.*D* genes. Lines represent introns, and gray boxes represent exons. White boxes represent 5’ or 3’ UTRs (untranslated regions). Triangles represent T-DNA inserts. (B) The catalytic domains of PP2C.D phosphatases were predicted by SMART (http://smart.embl-heidelberg.de). All T-DNA insertions are before or within the predicted catalytic domains.
(PDF)

S4 Fig RT-PCR analyses of *PP2C*.*D* transcripts in *pp2c*.*d* mutants. (A) RNAs were prepared from 7-day-old light-grown seedlings. Following reverse transcription, cDNAs were amplified by PCR (29 cycles) using primers spanning the T-DNA insertion site (S1 Table). (B) RT-PCR of the *pp2c*.*d2-2* allele (SALK_203806) was conducted as described above. All *pp2c*.*d* mutants appear to be null or severe knock-down alleles.
(PDF)

S5 Fig Shoot growth phenotypes of *pp2c*.*d* mutants. (A) and (B) Hypocotyl length of 8-day-old light-grown seedlings. *pp2c*.*d2-1* (WiscDsLox493G12) referred to as *d2* was used for generating various higher order *pp2c*.*d* mutants. Error bars = SEM (n = 41–52) **(**A) or SD (n = 20–26) (B). (C) Hypocotyl length of 7-day-old light-grown seedlings. Error bars = SD (n = 15). Two independent native promoter::PP2C.D(2, 5 or 6)-GFP lines were assessed for complementation of the *pp2c*.*d2/5/6* long hypocotyl phenotype. All three transgenes rescued the long hypocotyl phenotype of the *pp2c*.*d2/5/6* mutant. (D) Fifty-one-day-old plants and measurements of total plant height. Scale bar = 4 cm. Error bars = SD (n = 17–18). (E) Siliques and measurements of silique length. Scale bar = 4 mm. Error bars = SD (n = 92–94). Different letters above the bars indicate significant differences (P < 0.05).
(PDF)

S6 Fig PP2C.D phosphatases interact with SAUR19 in yeast two-hybrid assays. **(**A) Yeast two-hybrid assays examining PP2C.D and SAUR19 protein interactions. pACT2-PP2C.D and pBTM116-SAUR19 plasmids were co-transformed into yeast strain L40ccU3, plated onto appropriate selection media, and grown at room temperature for 3 to 6 d. Interactions were detected with all family members except PP2C.D7 and PP2C.D9. (B) Western blot analyses of PP2C.D1-HA, PP2C.D7-HA, and PP2C.D9-HA protein expression in yeast detected by an anti-HA antibody. Protein extracts were prepared from three independent yeast colonies.
(PDF)

S7 Fig PP2C.D phosphatases interact with plasma membrane H^+^-ATPases in *Nicotiana benthamiana* leaf epidermal cells. BiFC assays detecting PP2C.D and AHA2 protein interactions in *Nicotiana benthamiana* leaves.
(PDF)

S8 Fig Expression of PP2C.D5-HA protein complements the hypocotyl growth phenotype of *pp2c*.*d5* seedlings. (A) Hypocotyl length of 8-day-old seedlings grown under 30 μE m^-2^ s^-1^ light. Error bars = SEM (n = 33–58). Different letters above the bars indicate significant differences (P < 0.05). (B) Western blot analyses of PP2C.D5-HA protein expression. Twenty-five micrograms of total proteins were loaded. PP2C.D5-HA and the SEC12 loading control were detected by anti-HA and anti-SEC12 antibodies, respectively.
(PDF)

S9 Fig Expression of *PP2C*.*D-GUS* reporters in seedlings shifted to high temperature. (A) Four-day-old light-grown seedlings grown at 20 ^o^C were shifted to 28 ^o^C for 1 d and stained for β-glucuronidase activity. (B) Two-day-old light-grown seedlings grown at 20 ^o^C were shifted to 28 ^o^C for 5 d and stained for β-glucuronidase activity. Images on the left side of each panel depict GUS staining patterns of seedlings maintained at 20 ^o^C over the duration of the experiment. GUS staining was performed at 37 ^o^C overnight.
(PDF)

S1 Table PCR primers used in this study. (PDF)

## References

[1] Mironova V, Teale W, Shahriari M, Dawson J, Palme K. The Systems Biology of Auxin in Developing Embryos. Trends Plant Sci. 2017;22(3):225–35. Epub 2017/01/25. doi: 10.1016/j.tplants.2016.11.010 . 28131745 10.1016/j.tplants.2016.11.010

[2] Choe G, Lee JY. Push-pull strategy in the regulation of postembryonic root development. Curr Opin Plant Biol. 2017;35:158–64. Epub 2017/01/04. doi: 10.1016/j.pbi.2016.12.005 . 28063383 10.1016/j.pbi.2016.12.005

[3] Sato EM, Hijazi H, Bennett MJ, Vissenberg K, Swarup R. New insights into root gravitropic signalling. J Exp Bot. 2015;66(8):2155–65. Epub 2014/12/29. doi: 10.1093/jxb/eru515 ; PubMed Central PMCID: PMCPMC4986716. 25547917 10.1093/jxb/eru515 PMC4986716

[4] Sluis A, Hake S. Organogenesis in plants: initiation and elaboration of leaves. Trends Genet. 2015;31(6):300–6. Epub 2015/05/20. doi: 10.1016/j.tig.2015.04.004 . 26003219 10.1016/j.tig.2015.04.004

[5] Weijers D, Wagner D. Transcriptional Responses to the Auxin Hormone. Annu Rev Plant Biol. 2016;67:539–74. Epub 2016/02/22. doi: 10.1146/annurev-arplant-043015-112122 . 26905654 10.1146/annurev-arplant-043015-112122

[6] de Wit M, Galvão VC, Fankhauser C. Light-Mediated Hormonal Regulation of Plant Growth and Development. Annu Rev Plant Biol. 2016;67:513–37. Epub 2016/02/22. doi: 10.1146/annurev-arplant-043015-112252 . 26905653 10.1146/annurev-arplant-043015-112252

[7] Soyars CL, James SR, Nimchuk ZL. Ready, aim, shoot: stem cell regulation of the shoot apical meristem. Curr Opin Plant Biol. 2016;29:163–8. Epub 2016/01/22. doi: 10.1016/j.pbi.2015.12.002 . 26803586 10.1016/j.pbi.2015.12.002

[8] Song S, Qi T, Huang H, Xie D. Regulation of stamen development by coordinated actions of jasmonate, auxin, and gibberellin in Arabidopsis. Mol Plant. 2013;6(4):1065–73. Epub 2013/03/29. doi: 10.1093/mp/sst054 . 23543439 10.1093/mp/sst054

[9] Marsch-Martínez N, de Folter S. Hormonal control of the development of the gynoecium. Curr Opin Plant Biol. 2016;29:104–14. Epub 2016/01/19. doi: 10.1016/j.pbi.2015.12.006 . 26799132 10.1016/j.pbi.2015.12.006

[10] Perrot-Rechenmann C. Cellular responses to auxin: division versus expansion. Cold Spring Harb Perspect Biol. 2010;2(5):a001446 Epub 2010/04/07. doi: 10.1101/cshperspect.a001446 ; PubMed Central PMCID: PMCPMC2857164. 20452959 10.1101/cshperspect.a001446 PMC2857164

[11] Takatsuka H, Umeda M. Hormonal control of cell division and elongation along differentiation trajectories in roots. J Exp Bot. 2014;65(10):2633–43. Epub 2014/01/28. doi: 10.1093/jxb/ert485 . 24474807 10.1093/jxb/ert485

[12] Gray WM, Kepinski S, Rouse D, Leyser O, Estelle M. Auxin regulates SCF^TIR1^-dependent degradation of AUX/IAA proteins. Nature. 2001;414(6861):271–6. doi: 10.1038/35104500 . 11713520 10.1038/35104500

[13] Lavy M, Estelle M. Mechanisms of auxin signaling. Development. 2016;143(18):3226–9. doi: 10.1242/dev.131870 ; PubMed Central PMCID: PMCPMC5047657. 27624827 10.1242/dev.131870 PMC5047657

[14] Ren H, Gray WM. SAUR Proteins as Effectors of Hormonal and Environmental Signals in Plant Growth. Mol Plant. 2015;8(8):1153–64. Epub 2015/05/15. doi: 10.1016/j.molp.2015.05.003 ; PubMed Central PMCID: PMCPMC5124491. 25983207 10.1016/j.molp.2015.05.003 PMC5124491

[15] Rayle DL, Cleland RE. The Acid Growth Theory of auxin-induced cell elongation is alive and well. Plant Physiol. 1992;99(4):1271–4. ; PubMed Central PMCID: PMCPMC1080619. 11537886 10.1104/pp.99.4.1271 PMC1080619

[16] Dünser K, Kleine-Vehn J. Differential growth regulation in plants-the acid growth balloon theory. Curr Opin Plant Biol. 2015;28:55–9. Epub 2015/10/24. doi: 10.1016/j.pbi.2015.08.009 . 26454696 10.1016/j.pbi.2015.08.009

[17] Takahashi K, Hayashi K, Kinoshita T. Auxin activates the plasma membrane H^+^-ATPase by phosphorylation during hypocotyl elongation in Arabidopsis. Plant Physiol. 2012;159(2):632–41. Epub 2012/04/05. doi: 10.1104/pp.112.196428 ; PubMed Central PMCID: PMCPMC3375930. 22492846 10.1104/pp.112.196428 PMC3375930

[18] Spartz AK, Ren H, Park MY, Grandt KN, Lee SH, Murphy AS, et al SAUR Inhibition of PP2C-D Phosphatases Activates Plasma Membrane H^+^-ATPases to Promote Cell Expansion in Arabidopsis. Plant Cell. 2014;26(5):2129–42. Epub 2014/05/23. doi: 10.1105/tpc.114.126037 ; PubMed Central PMCID: PMCPMC4079373. 24858935 10.1105/tpc.114.126037 PMC4079373

[19] Spartz AK, Lor VS, Ren H, Olszewski NE, Miller ND, Wu G, et al Constitutive Expression of Arabidopsis *SMALL AUXIN UP RNA19* (*SAUR19*) in Tomato Confers Auxin-Independent Hypocotyl Elongation. Plant Physiol. 2017;173(2):1453–62. Epub 2016/12/20. doi: 10.1104/pp.16.01514 . 27999086 10.1104/pp.16.01514 PMC5291034

[20] Fendrych M, Leung J, Friml J. TIR1/AFB-Aux/IAA auxin perception mediates rapid cell wall acidification and growth of Arabidopsis hypocotyls. Elife. 2016;5 Epub 2016/09/14. doi: 10.7554/eLife.19048 ; PubMed Central PMCID: PMCPMC5045290. 27627746 10.7554/eLife.19048 PMC5045290

[21] Uchida N, Takahashi K, Iwasaki R, Yamada R, Yoshimura M, Endo TA, et al Chemical hijacking of auxin signaling with an engineered auxin-TIR1 pair. Nat Chem Biol. 2018;14(3):299–305. Epub 2018/01/22. doi: 10.1038/nchembio.2555 ; PubMed Central PMCID: PMCPMC5812785. 29355850 10.1038/nchembio.2555 PMC5812785

[22] Fuchs S, Grill E, Meskiene I, Schweighofer A. Type 2C protein phosphatases in plants. FEBS J. 2013;280(2):681–93. Epub 2012/07/17. doi: 10.1111/j.1742-4658.2012.08670.x . 22726910 10.1111/j.1742-4658.2012.08670.x

[23] Shi Y. Serine/threonine phosphatases: mechanism through structure. Cell. 2009;139(3):468–84. doi: 10.1016/j.cell.2009.10.006 . 19879837 10.1016/j.cell.2009.10.006

[24] Sentandreu M, Martín G, González-Schain N, Leivar P, Soy J, Tepperman JM, et al Functional profiling identifies genes involved in organ-specific branches of the PIF3 regulatory network in Arabidopsis. Plant Cell. 2011;23(11):3974–91. doi: 10.1105/tpc.111.088161 ; PubMed Central PMCID: PMCPMC3246323. 22108407 10.1105/tpc.111.088161 PMC3246323

[25] Xiao D, Cui Y, Xu F, Xu X, Gao G, Wang Y, et al SENESCENCE-SUPPRESSED PROTEIN PHOSPHATASE Directly Interacts with the Cytoplasmic Domain of SENESCENCE-ASSOCIATED RECEPTOR-LIKE KINASE and Negatively Regulates Leaf Senescence in Arabidopsis. Plant Physiol. 2015;169(2):1275–91. doi: 10.1104/pp.15.01112 ; PubMed Central PMCID: PMCPMC4587474. 26304848 10.1104/pp.15.01112 PMC4587474

[26] Couto D, Niebergall R, Liang X, Bücherl CA, Sklenar J, Macho AP, et al The Arabidopsis Protein Phosphatase PP2C38 Negatively Regulates the Central Immune Kinase BIK1. PLoS Pathog. 2016;12(8):e1005811 doi: 10.1371/journal.ppat.1005811 ; PubMed Central PMCID: PMCPMC4975489. 27494702 10.1371/journal.ppat.1005811 PMC4975489

[27] Winter D, Vinegar B, Nahal H, Ammar R, Wilson GV, Provart NJ. An "Electronic Fluorescent Pictograph" browser for exploring and analyzing large-scale biological data sets. PLoS One. 2007;2(8):e718 Epub 2007/08/08. doi: 10.1371/journal.pone.0000718 ; PubMed Central PMCID: PMCPMC1934936. 17684564 10.1371/journal.pone.0000718 PMC1934936

[28] Tovar-Mendez A, Miernyk JA, Hoyos E, Randall DD. A functional genomic analysis of *Arabidopsis thaliana* PP2C clade D. Protoplasma. 2014;251(1):265–71. doi: 10.1007/s00709-013-0526-7 . 23832523 10.1007/s00709-013-0526-7

[29] Nemhauser JL, Hong F, Chory J. Different plant hormones regulate similar processes through largely nonoverlapping transcriptional responses. Cell. 2006;126(3):467–75. doi: 10.1016/j.cell.2006.05.050 . 16901781 10.1016/j.cell.2006.05.050

[30] Gendreau E, Traas J, Desnos T, Grandjean O, Caboche M, Höfte H. Cellular basis of hypocotyl growth in *Arabidopsis thaliana*. Plant Physiol. 1997;114(1):295–305. ; PubMed Central PMCID: PMCPMC158305. 9159952 10.1104/pp.114.1.295 PMC158305

[31] Haruta M, Sussman MR. The effect of a genetically reduced plasma membrane protonmotive force on vegetative growth of Arabidopsis. Plant Physiol. 2012;158(3):1158–71. Epub 2012/01/03. doi: 10.1104/pp.111.189167 ; PubMed Central PMCID: PMCPMC3291248. 22214817 10.1104/pp.111.189167 PMC3291248

[32] Hayashi Y, Nakamura S, Takemiya A, Takahashi Y, Shimazaki K, Kinoshita T. Biochemical characterization of in vitro phosphorylation and dephosphorylation of the plasma membrane H^+^-ATPase. Plant Cell Physiol. 2010;51(7):1186–96. Epub 2010/06/01. doi: 10.1093/pcp/pcq078 . 20516032 10.1093/pcp/pcq078

[33] Fuglsang AT, Visconti S, Drumm K, Jahn T, Stensballe A, Mattei B, et al Binding of 14-3-3 protein to the plasma membrane H^+^-ATPase AHA2 involves the three C-terminal residues Tyr^946^-Thr-Val and requires phosphorylation of Thr^947^. J Biol Chem. 1999;274(51):36774–80. . 10593986 10.1074/jbc.274.51.36774

[34] Chae K, Isaacs CG, Reeves PH, Maloney GS, Muday GK, Nagpal P, et al Arabidopsis *SMALL AUXIN UP RNA63* promotes hypocotyl and stamen filament elongation. Plant J. 2012;71(4):684–97. Epub 2012/06/14. doi: 10.1111/j.1365-313X.2012.05024.x . 22507274 10.1111/j.1365-313X.2012.05024.x

[35] van Mourik H, van Dijk ADJ, Stortenbeker N, Angenent GC, Bemer M. Divergent regulation of Arabidopsis *SAUR* genes: a focus on the *SAUR10*-clade. BMC Plant Biol. 2017;17(1):245 Epub 2017/12/19. doi: 10.1186/s12870-017-1210-4 ; PubMed Central PMCID: PMCPMC5735953. 29258424 10.1186/s12870-017-1210-4 PMC5735953

[36] Spartz AK, Lee SH, Wenger JP, Gonzalez N, Itoh H, Inzé D, et al The *SAUR19* subfamily of *SMALL AUXIN UP RNA* genes promote cell expansion. Plant J. 2012;70(6):978–90. doi: 10.1111/j.1365-313X.2012.04946.x ; PubMed Central PMCID: PMCPMC3481998. 22348445 10.1111/j.1365-313X.2012.04946.x PMC3481998

[37] McClure BA, Guilfoyle T. Rapid redistribution of auxin-regulated RNAs during gravitropism. Science. 1989;243:91–3. . 11540631 10.1126/science.11540631

[38] Atamian HS, Creux NM, Brown EA, Garner AG, Blackman BK, Harmer SL. Circadian regulation of sunflower heliotropism, floral orientation, and pollinator visits. Science. 2016;353(6299):587–90. doi: 10.1126/science.aaf9793 . 27493185 10.1126/science.aaf9793

[39] Esmon CA, Tinsley AG, Ljung K, Sandberg G, Hearne LB, Liscum E. A gradient of auxin and auxin-dependent transcription precedes tropic growth responses. Proc Natl Acad Sci U S A. 2006;103(1):236–41. Epub 2005/12/21. doi: 10.1073/pnas.0507127103 ; PubMed Central PMCID: PMCPMC1324985. 16371470 10.1073/pnas.0507127103 PMC1324985

[40] Fuglsang AT, Guo Y, Cuin TA, Qiu Q, Song C, Kristiansen KA, et al Arabidopsis protein kinase PKS5 inhibits the plasma membrane H^+^-ATPase by preventing interaction with 14-3-3 protein. Plant Cell. 2007;19(5):1617–34. Epub 2007/05/04. doi: 10.1105/tpc.105.035626 ; PubMed Central PMCID: PMCPMC1913743. 17483306 10.1105/tpc.105.035626 PMC1913743

[41] Schweighofer A, Hirt H, Meskiene I. Plant PP2C phosphatases: emerging functions in stress signaling. Trends Plant Sci. 2004;9(5):236–43. doi: 10.1016/j.tplants.2004.03.007 . 15130549 10.1016/j.tplants.2004.03.007

[42] Chapman EJ, Greenham K, Castillejo C, Sartor R, Bialy A, Sun TP, et al Hypocotyl transcriptome reveals auxin regulation of growth-promoting genes through GA-dependent and -independent pathways. PLoS One. 2012;7(5):e36210 doi: 10.1371/journal.pone.0036210 ; PubMed Central PMCID: PMCPMC3348943. 22590525 10.1371/journal.pone.0036210 PMC3348943

[43] Franklin KA, Lee SH, Patel D, Kumar SV, Spartz AK, Gu C, et al Phytochrome-interacting factor 4 (PIF4) regulates auxin biosynthesis at high temperature. Proc Natl Acad Sci U S A. 2011;108(50):20231–5. doi: 10.1073/pnas.1110682108 ; PubMed Central PMCID: PMCPMC3250122. 22123947 10.1073/pnas.1110682108 PMC3250122

[44] Park JE, Kim YS, Yoon HK, Park CM. Functional characterization of a *small auxin-up RNA* gene in apical hook development in Arabidopsis. Plant Sci. 2007;172(1):150–7.

[45] Narsai R, Law SR, Carrie C, Xu L, Whelan J. In-depth temporal transcriptome profiling reveals a crucial developmental switch with roles for RNA processing and organelle metabolism that are essential for germination in Arabidopsis. Plant Physiol. 2011;157(3):1342–62. Epub 2011/09/09. doi: 10.1104/pp.111.183129 ; PubMed Central PMCID: PMCPMC3252162. 21908688 10.1104/pp.111.183129 PMC3252162

[46] Qiu T, Chen Y, Li M, Kong Y, Zhu Y, Han N, et al The tissue-specific and developmentally regulated expression patterns of the *SAUR41* subfamily of small auxin up RNA genes: potential implications. Plant Signal Behav. 2013;8(8). Epub 2013/06/10. doi: 10.4161/psb.25283 ; PubMed Central PMCID: PMCPMC3999058. 23759547 10.4161/psb.25283 PMC3999058

[47] Kong Y, Zhu Y, Gao C, She W, Lin W, Chen Y, et al Tissue-specific expression of *SMALL AUXIN UP RNA41* differentially regulates cell expansion and root meristem patterning in Arabidopsis. Plant Cell Physiol. 2013;54(4):609–21. Epub 2013/02/08. doi: 10.1093/pcp/pct028 . 23396598 10.1093/pcp/pct028

[48] Cheng Y, Dai X, Zhao Y. Auxin biosynthesis by the YUCCA flavin monooxygenases controls the formation of floral organs and vascular tissues in Arabidopsis. Genes Dev. 2006;20(13):1790–9. doi: 10.1101/gad.1415106 ; PubMed Central PMCID: PMCPMC1522075. 16818609 10.1101/gad.1415106 PMC1522075

[49] Cecchetti V, Altamura MM, Falasca G, Costantino P, Cardarelli M. Auxin regulates Arabidopsis anther dehiscence, pollen maturation, and filament elongation. Plant Cell. 2008;20(7):1760–74. Epub 2008/07/15. doi: 10.1105/tpc.107.057570 ; PubMed Central PMCID: PMCPMC2518247. 18628351 10.1105/tpc.107.057570 PMC2518247

[50] Nagpal P, Ellis CM, Weber H, Ploense SE, Barkawi LS, Guilfoyle TJ, et al Auxin response factors ARF6 and ARF8 promote jasmonic acid production and flower maturation. Development. 2005;132(18):4107–18. Epub 2005/08/17. doi: 10.1242/dev.01955 . 16107481 10.1242/dev.01955

[51] Béziat C, Kleine-Vehn J. The Road to Auxin-Dependent Growth Repression and Promotion in Apical Hooks. Curr Biol. 2018;28(8):R519–R25. doi: 10.1016/j.cub.2018.01.069 . 29689235 10.1016/j.cub.2018.01.069

[52] Li Y, Hagen G, Guilfoyle TJ. An Auxin-Responsive Promoter Is Differentially Induced by Auxin Gradients during Tropisms. Plant Cell. 1991;3(11):1167–75. doi: 10.1105/tpc.3.11.1167 ; PubMed Central PMCID: PMCPMC160083. 12324587 10.1105/tpc.3.11.1167 PMC160083

[53] Gee MA, Hagen G, Guilfoyle TJ. Tissue-specific and organ-specific expression of soybean auxin-responsive transcripts GH3 and SAURs. Plant Cell. 1991;3(4):419–30. doi: 10.1105/tpc.3.4.419 ; PubMed Central PMCID: PMCPMC160011. 1840920 10.1105/tpc.3.4.419 PMC160011

[54] Hu L MZ, Zang A, Chen H, Dou X, Jin J, Cai W. Microarray analyses and comparisons of upper or lower flanks of rice shoot base preceding gravitropic bending. PLoS One. 2013;8(9):e74646 doi: 10.1371/journal.pone.0074646 24040303 10.1371/journal.pone.0074646 PMC3764065

[55] Taniguchi M, Nakamura M, Tasaka M, Morita MT. Identification of gravitropic response indicator genes in Arabidopsis inflorescence stems. Plant Signal Behav. 2014;9(9):e29570 doi: 10.4161/psb.29570 ; PubMed Central PMCID: PMCPMC4203507. 25763694 10.4161/psb.29570 PMC4203507

[56] Gray WM, Ostin A, Sandberg G, Romano CP, Estelle M. High temperature promotes auxin-mediated hypocotyl elongation in Arabidopsis. Proc Natl Acad Sci U S A. 1998;95(12):7197–202. ; PubMed Central PMCID: PMCPMC22781. 9618562 10.1073/pnas.95.12.7197 PMC22781

[57] Quint M, Delker C, Franklin KA, Wigge PA, Halliday KJ, van Zanten M. Molecular and genetic control of plant thermomorphogenesis. Nat Plants. 2016;2:15190 Epub 2016/01/06. doi: 10.1038/nplants.2015.190 . 27250752 10.1038/nplants.2015.190

[58] Nakagawa T, Kurose T, Hino T, Tanaka K, Kawamukai M, Niwa Y, et al Development of series of gateway binary vectors, pGWBs, for realizing efficient construction of fusion genes for plant transformation. J Biosci Bioeng. 2007;104(1):34–41. doi: 10.1263/jbb.104.34 . 17697981 10.1263/jbb.104.34

[59] Earley KW, Haag JR, Pontes O, Opper K, Juehne T, Song K, et al Gateway-compatible vectors for plant functional genomics and proteomics. Plant J. 2006;45(4):616–29. doi: 10.1111/j.1365-313X.2005.02617.x . 16441352 10.1111/j.1365-313X.2005.02617.x

[60] Karimi M, Inzé D, Depicker A. GATEWAY vectors for Agrobacterium-mediated plant transformation. Trends Plant Sci. 2002;7(5):193–5. . 11992820 10.1016/s1360-1385(02)02251-3

[61] Clough SJ, Bent AF. Floral dip: a simplified method for Agrobacterium-mediated transformation of Arabidopsis thaliana. Plant J. 1998;16(6):735–43. . 10069079 10.1046/j.1365-313x.1998.00343.x

[62] Ito H, Gray WM. A gain-of-function mutation in the Arabidopsis pleiotropic drug resistance transporter PDR9 confers resistance to auxinic herbicides. Plant Physiol. 2006;142(1):63–74. Epub 2006/07/28. doi: 10.1104/pp.106.084533 ; PubMed Central PMCID: PMCPMC1557603. 16877699 10.1104/pp.106.084533 PMC1557603

[63] Weber H, Bernhardt A, Dieterle M, Hano P, Mutlu A, Estelle M, et al Arabidopsis AtCUL3a and AtCUL3b form complexes with members of the BTB/POZ-MATH protein family. Plant Physiol. 2005;137(1):83–93. Epub 2004/12/23. doi: 10.1104/pp.104.052654 ; PubMed Central PMCID: PMCPMC548840. 15618422 10.1104/pp.104.052654 PMC548840

[64] Walter M, Chaban C, Schütze K, Batistic O, Weckermann K, Näke C, et al Visualization of protein interactions in living plant cells using bimolecular fluorescence complementation. Plant J. 2004;40(3):428–38. doi: 10.1111/j.1365-313X.2004.02219.x . 15469500 10.1111/j.1365-313X.2004.02219.x

[65] Schütze K, Harter K, Chaban C. Bimolecular fluorescence complementation (BiFC) to study protein-protein interactions in living plant cells. Methods Mol Biol. 2009;479:189–202. doi: 10.1007/978-1-59745-289-2_12 . 19083187 10.1007/978-1-59745-289-2_12

