# Supplementary material for: A subset of plasma membrane-localized PP2C.D phosphatases negatively regulate SAUR-mediated cell expansion in Arabidopsis
Source: PLoS Genet. 2018 Jun 13;14(6):e1007455. doi: 10.1371/journal.pgen.1007455 (PMC6016943; doi:10.1371/journal.pgen.1007455)
Supplement: S1 Fig — β-glucuronidase staining patterns of (A) 2-week-old light-grown plants, (B) flowers, and (C) siliques. GUS staining was performed at 37 oC for 24 h. Scale bars = 2 mm (A and C) or 1 mm (B). (PDF) [file pgen.1007455.s001.pdf]

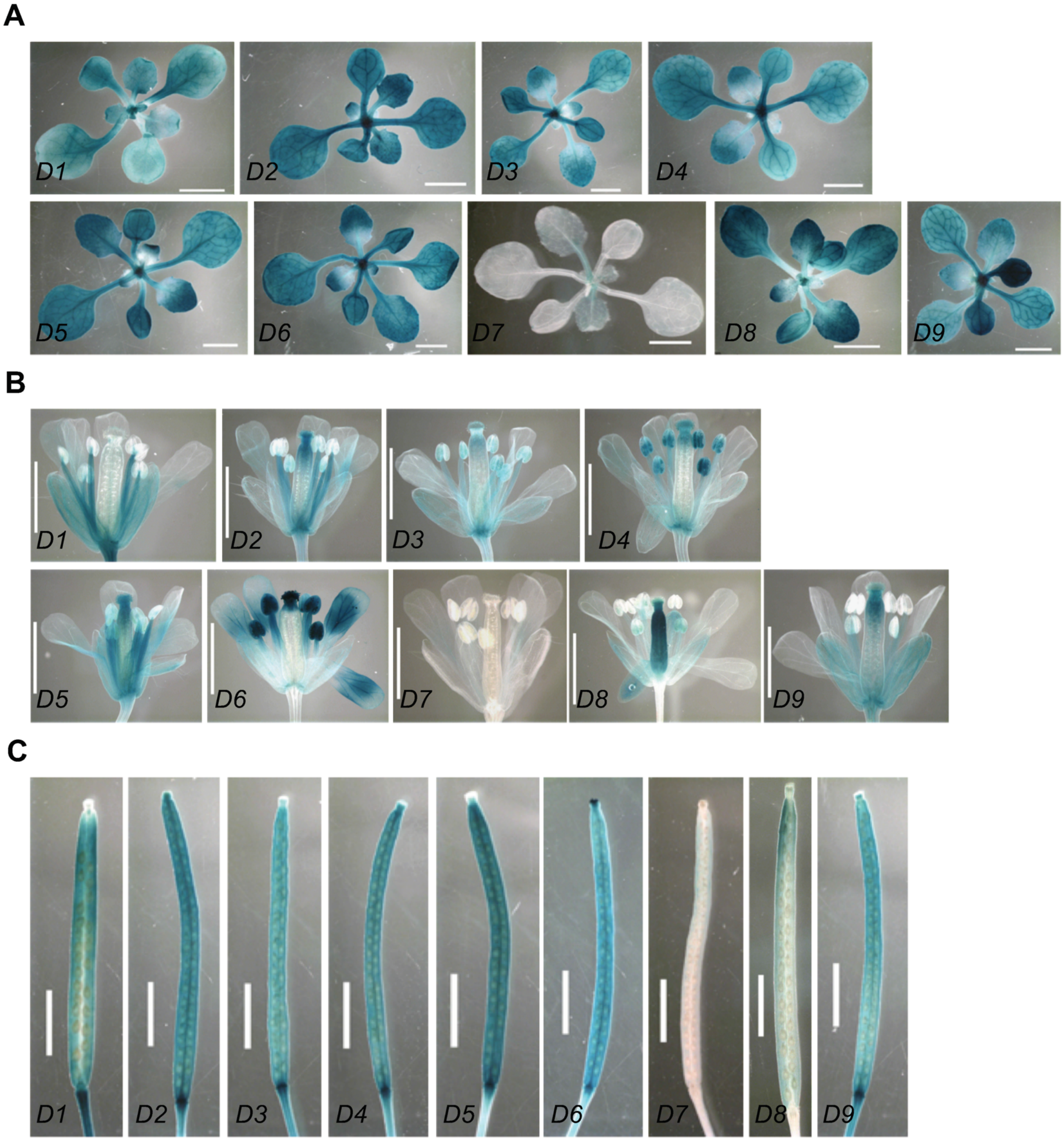

S1 Fig. Expression patterns of *PP2C.D-GUS* reporters. β-glucuronidase staining patterns of (A) 2-week-old light-grown plants, (B) flowers, and (C) siliques. GUS staining was performed at 37 °C for 24 h. Scale bars = 2 mm (A and C) or 1 mm (B).
