# Supplementary material for: A subset of plasma membrane-localized PP2C.D phosphatases negatively regulate SAUR-mediated cell expansion in Arabidopsis
Source: PLoS Genet. 2018 Jun 13;14(6):e1007455. doi: 10.1371/journal.pgen.1007455 (PMC6016943; doi:10.1371/journal.pgen.1007455)
Supplement: S3 Fig — (A) T-DNA insertion locations in PP2C.D genes. Lines represent introns, and gray boxes represent exons. White boxes represent 5’ or 3’ UTRs (untranslated regions). Triangles represent T-DNA inserts. (B) The catalytic domains of PP2C.D phosphatases were predicted by SMART (http://smart.embl-heidelberg.de). All T-DNA insertions are before or within the predicted catalytic domains. (PDF) [file pgen.1007455.s003.pdf]

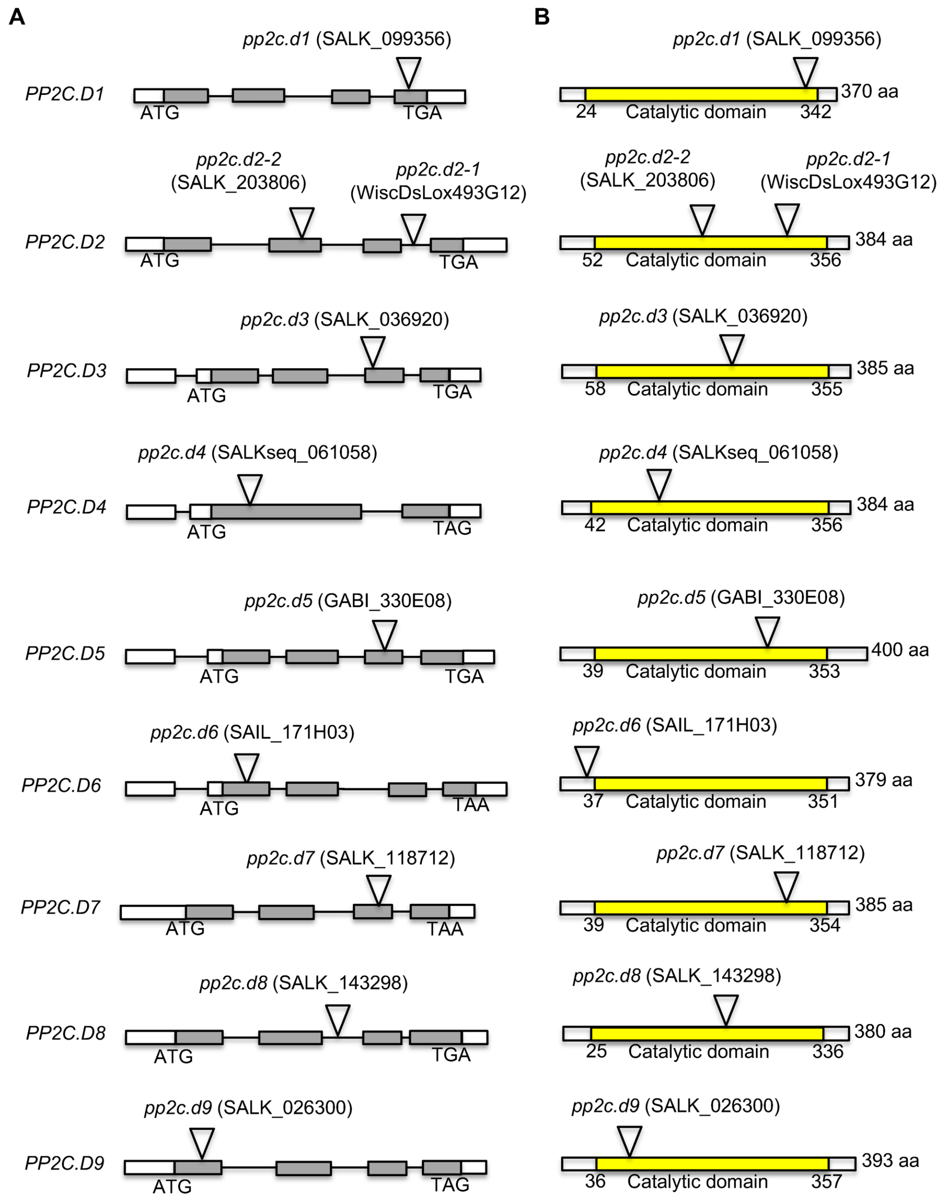

S3 Fig. *pp2c.d* T-DNA insertion mutants. (A) T-DNA insertion locations in *PP2C.D* genes. Lines represent introns, and gray boxes represent exons. White boxes represent 5' or 3' UTRs (untranslated regions). Triangles represent T-DNA inserts. (B) The catalytic domains of PP2C.D phosphatases were predicted by SMART (http://smart.embl-heidelberg.de). All T-DNA insertions are before or within the predicted catalytic domains.
