# Supplementary material for: A subset of plasma membrane-localized PP2C.D phosphatases negatively regulate SAUR-mediated cell expansion in Arabidopsis
Source: PLoS Genet. 2018 Jun 13;14(6):e1007455. doi: 10.1371/journal.pgen.1007455 (PMC6016943; doi:10.1371/journal.pgen.1007455)
Supplement: S4 Fig — (A) RNAs were prepared from 7-day-old light-grown seedlings. Following reverse transcription, cDNAs were amplified by PCR (29 cycles) using primers spanning the T-DNA insertion site (S1 Table). (B) RT-PCR of the pp2c.d2-2 allele (SALK_203806) was conducted as described above. All pp2c.d mutants appear to be null or severe knock-down alleles. (PDF) [file pgen.1007455.s004.pdf]

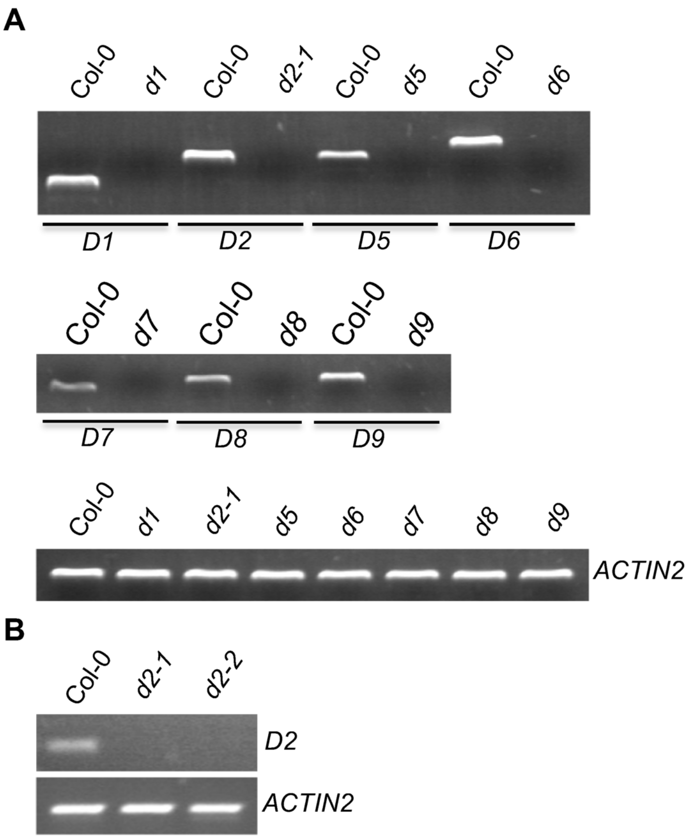

S4 Fig. RT-PCR analyses of *PP2C.D* transcripts in *pp2c.d* mutants. (A) RNAs were prepared from 7-day-old light-grown seedlings. Following reverse transcription, cDNAs were amplified by PCR (29 cycles) using primers spanning the T-DNA insertion site (Table S1). (B) RT-PCR of the *pp2c.d2-2* allele (SALK_203806) was conducted as described above. All *pp2c.d* mutants appear to be null or severe knock-down alleles.
