# Supplementary material for: A subset of plasma membrane-localized PP2C.D phosphatases negatively regulate SAUR-mediated cell expansion in Arabidopsis
Source: PLoS Genet. 2018 Jun 13;14(6):e1007455. doi: 10.1371/journal.pgen.1007455 (PMC6016943; doi:10.1371/journal.pgen.1007455)
Supplement: S5 Fig — (A) and (B) Hypocotyl length of 8-day-old light-grown seedlings. pp2c.d2-1 (WiscDsLox493G12) referred to as d2 was used for generating various higher order pp2c.d mutants. Error bars = SEM (n = 41–52) (A) or SD (n = 20–26) (B). (C) Hypocotyl length of 7-day-old light-grown seedlings. Error bars = SD (n = 15). Two independent native promoter::PP2C.D(2, 5 or 6)-GFP lines were assessed for complementation of the pp2c.d2/5/6 long hypocotyl phenotype. All three transgenes rescued the long hypocotyl phenotype of the pp2c.d2/5/6 mutant. (D) Fifty-one-day-old plants and measurements of total plant height. Scale bar = 4 cm. Error bars = SD (n = 17–18). (E) Siliques and measurements of silique length. Scale bar = 4 mm. Error bars = SD (n = 92–94). Different letters above the bars indicate significant differences (P < 0.05). (PDF) [file pgen.1007455.s005.pdf]

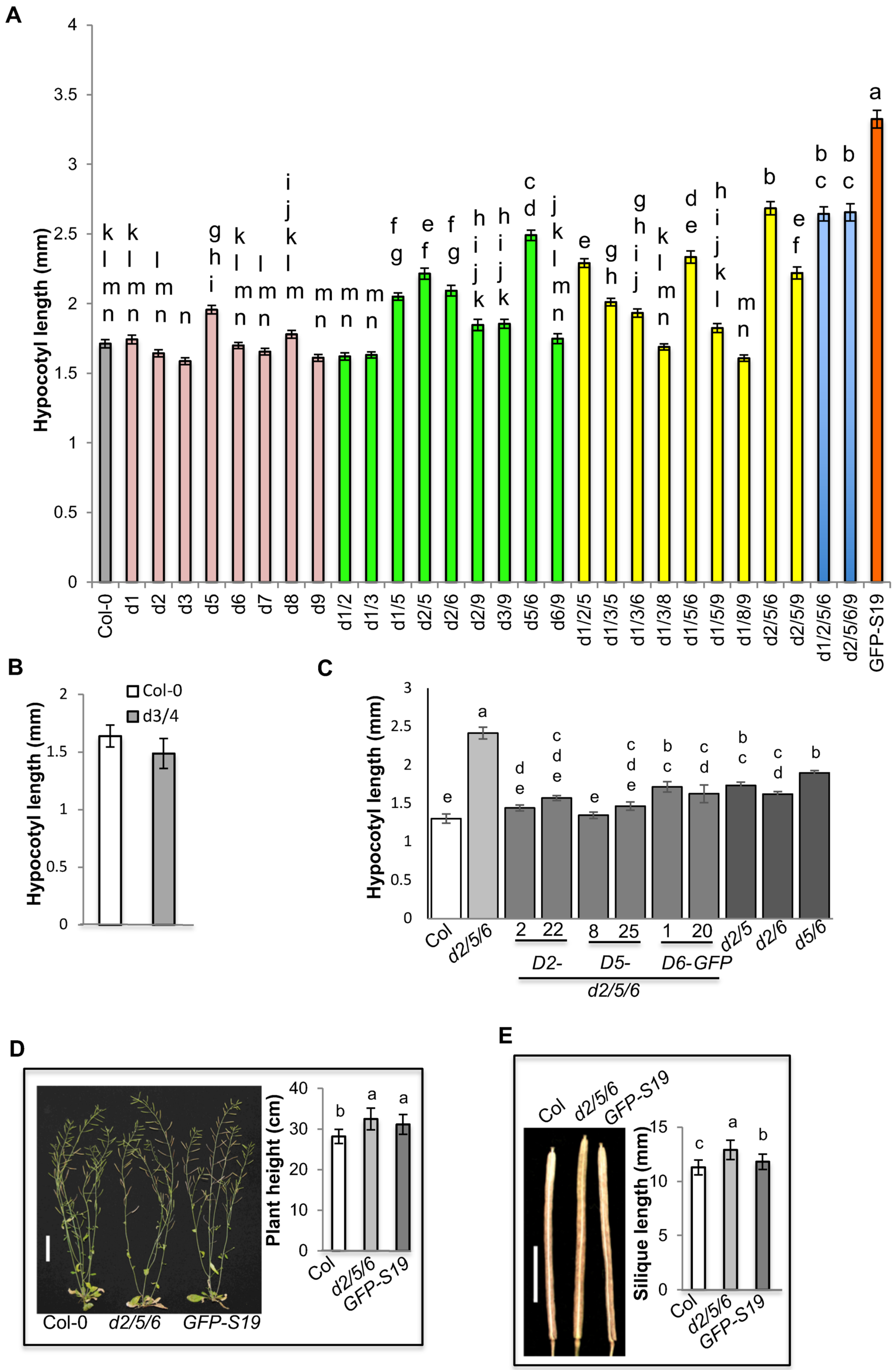

S5 Fig. Shoot growth phenotypes of *pp2c.d* mutants. (A) and (B) Hypocotyl length

of 8-day-old light-grown seedlings. *pp2c.d2-1* (WiscDsLox493G12) referred to as *d2* was used for generating various higher order *pp2c.d* mutants. Error bars = SEM (n = 41 - 52) (A) or SD (n = 20 - 26) (B). (C) Hypocotyl length of 7-day-old light-grown seedlings. Error bars = SD (n = 15). Two independent native promoter::PP2C.D(2, 5 or 6)-GFP lines were assessed for complementation of the *pp2c.d2/5/6* long hypocotyl phenotype. All three transgenes rescued the long hypocotyl phenotype of the *pp2c.d2/5/6* mutant. (D) Fifty-one-day-old plants and measurements of total plant height. Scale bar = 4 cm. Error bars = SD (n = 17 - 18). (E) Siliques and measurements of silique length. Scale bar = 4 mm. Error bars = SD (n = 92 - 94). Different letters above the bars indicate significant differences ($P < 0.05$).
