# Supplementary material for: A subset of plasma membrane-localized PP2C.D phosphatases negatively regulate SAUR-mediated cell expansion in Arabidopsis
Source: PLoS Genet. 2018 Jun 13;14(6):e1007455. doi: 10.1371/journal.pgen.1007455 (PMC6016943; doi:10.1371/journal.pgen.1007455)
Supplement: S6 Fig — (A) Yeast two-hybrid assays examining PP2C.D and SAUR19 protein interactions. pACT2-PP2C.D and pBTM116-SAUR19 plasmids were co-transformed into yeast strain L40ccU3, plated onto appropriate selection media, and grown at room temperature for 3 to 6 d. Interactions were detected with all family members except PP2C.D7 and PP2C.D9. (B) Western blot analyses of PP2C.D1-HA, PP2C.D7-HA, and PP2C.D9-HA protein expression in yeast detected by an anti-HA antibody. Protein extracts were prepared from three independent yeast colonies. (PDF) [file pgen.1007455.s006.pdf]

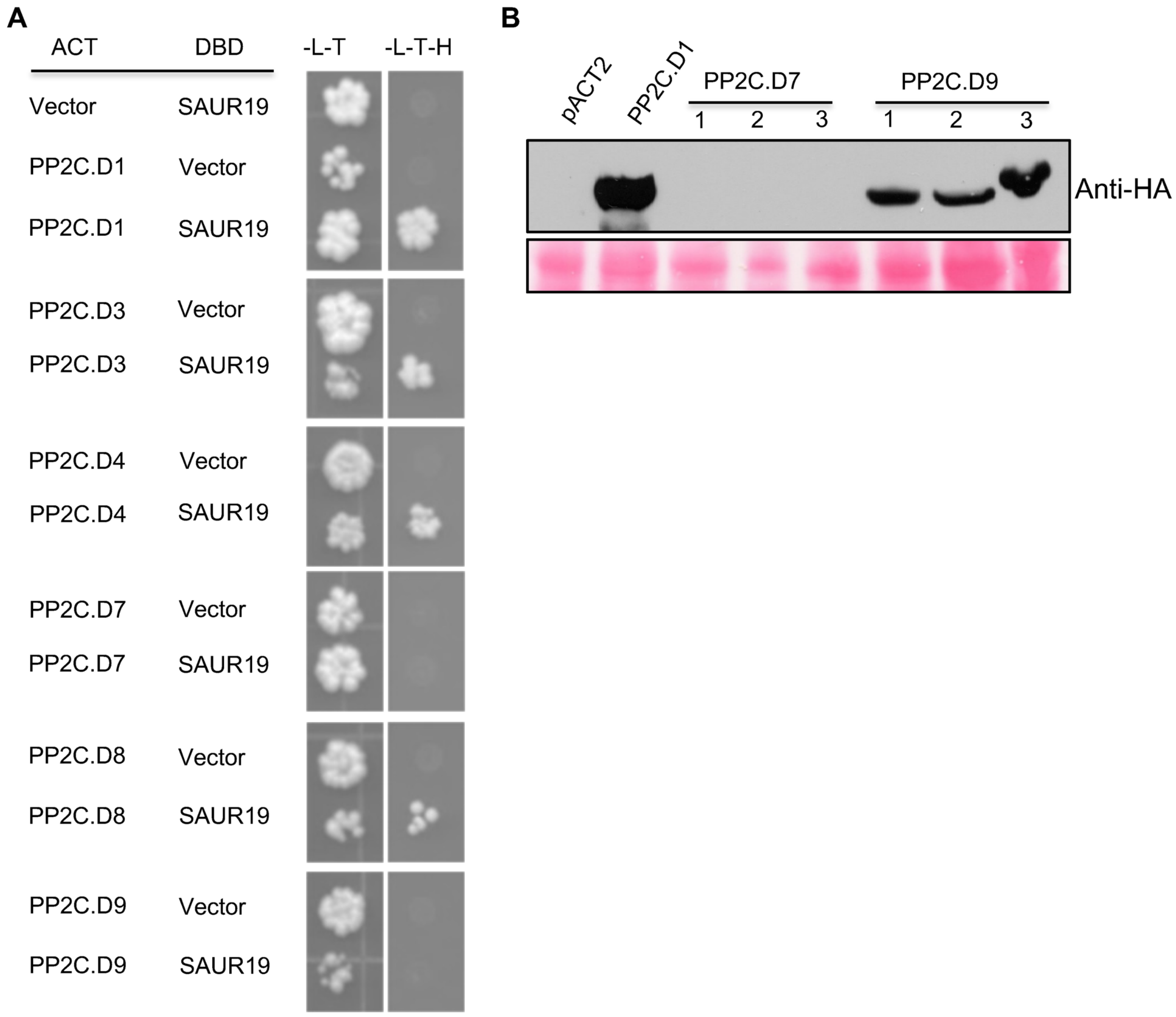

S6 Fig. PP2C.D phosphatases interact with SAUR19 in yeast two-hybrid assays. (A) Yeast two-hybrid assays examining PP2C.D and SAUR19 protein interactions. pACT2-PP2C.D and pBTM116-SAUR19 plasmids were co-transformed into yeast strain L40ccU3, plated onto appropriate selection media, and grown at room temperature for 3 to 6 d. Interactions were detected with all family members except PP2C.D7 and PP2C.D9. (B) Western blot analyses of PP2C.D1-HA, PP2C.D7-HA, and PP2C.D9-HA protein expression in yeast detected by an anti-HA antibody. Protein extracts were prepared from three independent yeast colonies.
