# Supplementary material for: A subset of plasma membrane-localized PP2C.D phosphatases negatively regulate SAUR-mediated cell expansion in Arabidopsis
Source: PLoS Genet. 2018 Jun 13;14(6):e1007455. doi: 10.1371/journal.pgen.1007455 (PMC6016943; doi:10.1371/journal.pgen.1007455)
Supplement: S7 Fig — BiFC assays detecting PP2C.D and AHA2 protein interactions in Nicotiana benthamiana leaves. (PDF) [file pgen.1007455.s007.pdf]

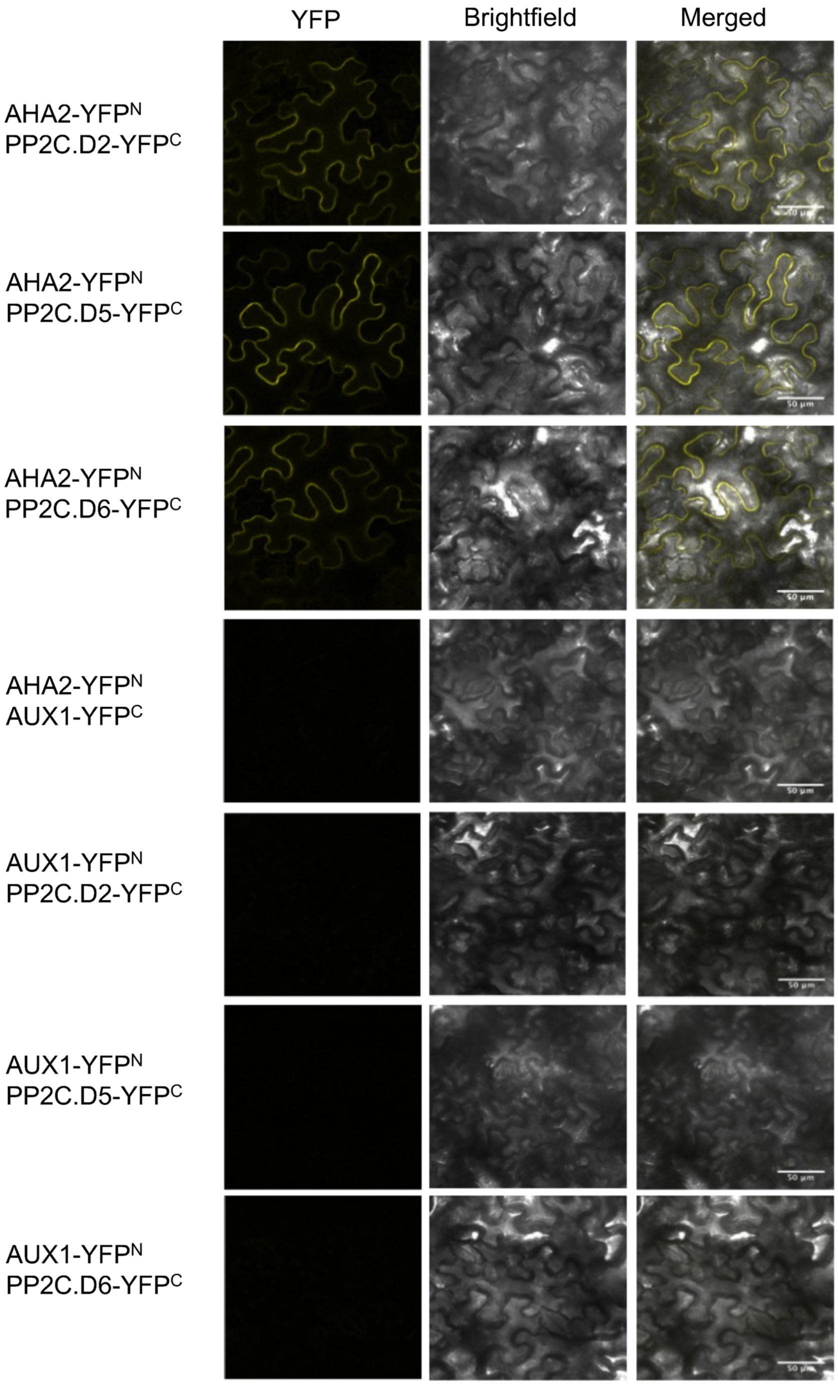

S7 Fig. PP2C.D phosphatases interact with plasma membrane H^+-ATPases in *Nicotiana benthamiana* leaf epidermal cells. BiFC assays detecting PP2C.D and AHA2 protein interactions in *Nicotiana benthamiana* leaves.
