# Supplementary material for: A subset of plasma membrane-localized PP2C.D phosphatases negatively regulate SAUR-mediated cell expansion in Arabidopsis
Source: PLoS Genet. 2018 Jun 13;14(6):e1007455. doi: 10.1371/journal.pgen.1007455 (PMC6016943; doi:10.1371/journal.pgen.1007455)
Supplement: S8 Fig — (A) Hypocotyl length of 8-day-old seedlings grown under 30 μE m-2 s-1 light. Error bars = SEM (n = 33–58). Different letters above the bars indicate significant differences (P < 0.05). (B) Western blot analyses of PP2C.D5-HA protein expression. Twenty-five micrograms of total proteins were loaded. PP2C.D5-HA and the SEC12 loading control were detected by anti-HA and anti-SEC12 antibodies, respectively. (PDF) [file pgen.1007455.s008.pdf]

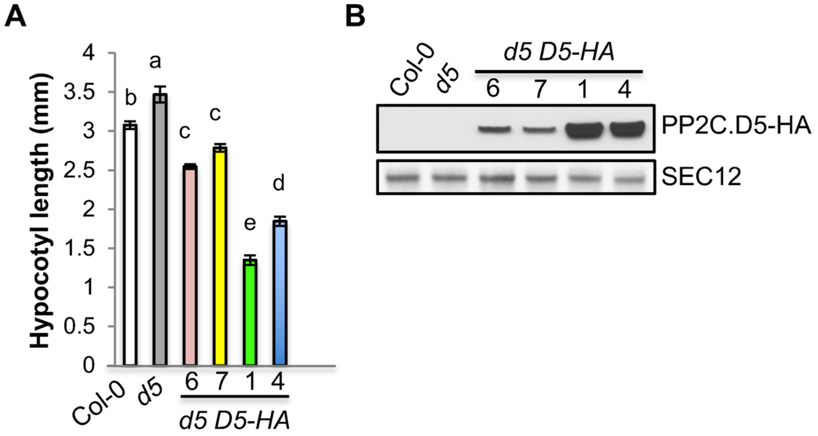

S8 Fig. Expression of PP2C.D5-HA protein complements the hypocotyl growth phenotype of *pp2c.d5* seedlings. (A) Hypocotyl length of 8-day-old seedlings grown under 30 µE m^{-2} s^{-1} light. Error bars = SEM (n = 33 - 58). Different letters above the bars indicate significant differences ($P < 0.05$). (B) Western blot analyses of PP2C.D5-HA protein expression. Twenty-five micrograms of total proteins were loaded. PP2C.D5-HA and the SEC12 loading control were detected by anti-HA and anti-SEC12 antibodies, respectively.
