# Supplementary material for: A subset of plasma membrane-localized PP2C.D phosphatases negatively regulate SAUR-mediated cell expansion in Arabidopsis
Source: PLoS Genet. 2018 Jun 13;14(6):e1007455. doi: 10.1371/journal.pgen.1007455 (PMC6016943; doi:10.1371/journal.pgen.1007455)
Supplement: S9 Fig — (A) Four-day-old light-grown seedlings grown at 20 oC were shifted to 28 oC for 1 d and stained for β-glucuronidase activity. (B) Two-day-old light-grown seedlings grown at 20 oC were shifted to 28 oC for 5 d and stained for β-glucuronidase activity. Images on the left side of each panel depict GUS staining patterns of seedlings maintained at 20 oC over the duration of the experiment. GUS staining was performed at 37 oC overnight. (PDF) [file pgen.1007455.s009.pdf]

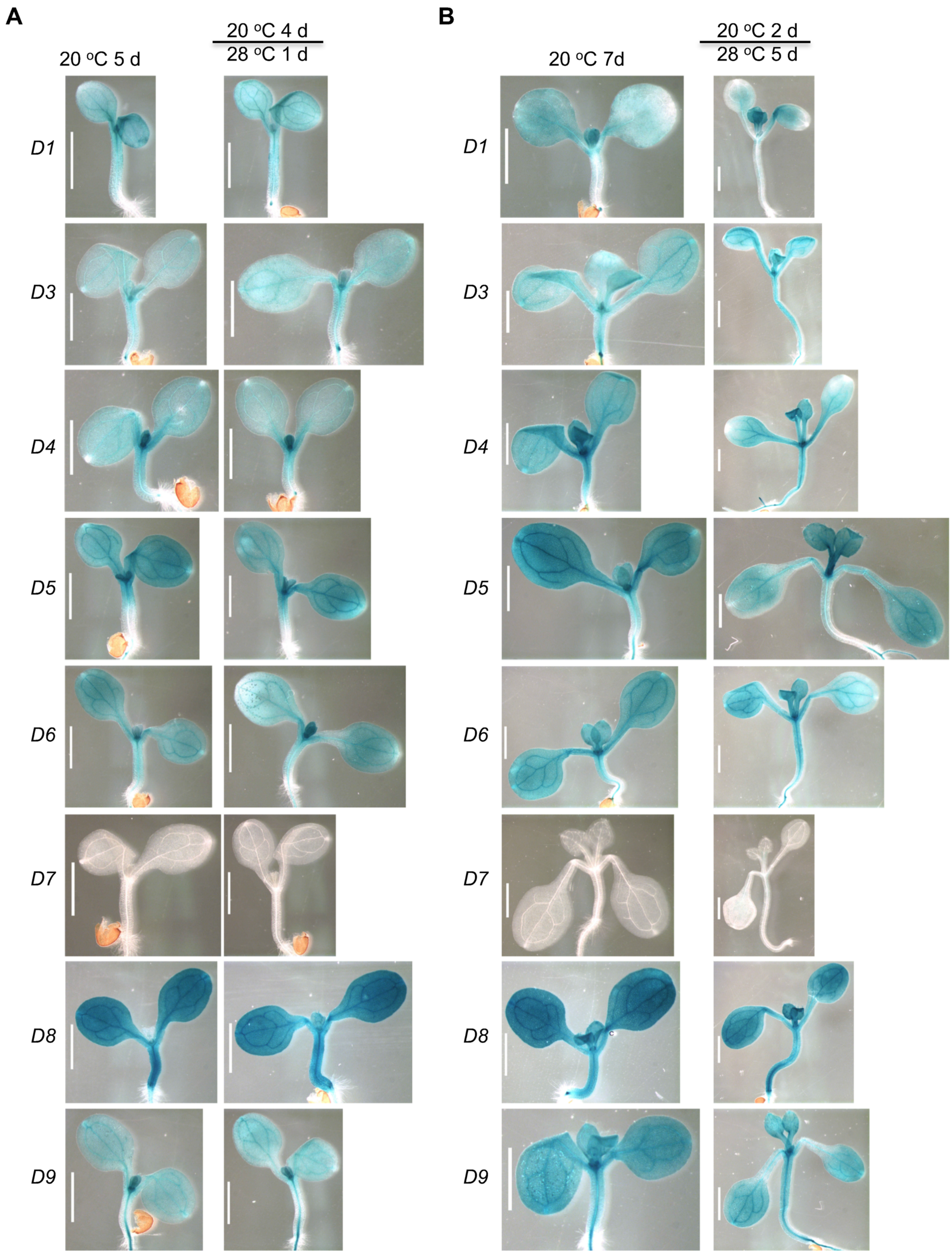

S9 Fig. Expression of *PP2C.D-GUS* reporters in seedlings shifted to high temperature.

(A) Four-day-old light-grown seedlings grown at 20 ^{o}C were shifted to 28 ^{o}C for 1 d and stained for β-glucuronidase activity. (B) Two-day-old light-grown seedlings grown at 20 ^{o}C were shifted to 28 ^{o}C for 5 d and stained for β-glucuronidase activity. Images on the left side of each panel depict GUS staining patterns of seedlings maintained at 20 ^{o}C over the duration of the experiment. GUS staining was performed at 37 ^{o}C overnight.
