# Supplementary material for: A subset of plasma membrane-localized PP2C.D phosphatases negatively regulate SAUR-mediated cell expansion in Arabidopsis
Source: PLoS Genet. 2018 Jun 13;14(6):e1007455. doi: 10.1371/journal.pgen.1007455 (PMC6016943; doi:10.1371/journal.pgen.1007455)
Supplement: S1 Table — (PDF) [file pgen.1007455.s010.pdf]

S1 Table. PCR primers used used in this study.

Primer names	Sequence	Length of *PP2C.D* promoter fragments for GUS/GFP reporters
PP2C.D1-FW1	CACCTCAGTTTGCGGTGATGGGTAC	4422 bp
PP2C.D1-RV1	CTTTCTCAGGTATGTAATCTTCG	
PP2C.D1-RV2	TGATGTTGAATGCATCGGGTATC	
PP2C.D2-FW1	CACCTGAGCTTTTCTTTTATGCTTAACG	1013 bp
PP2C.D2-RV1	ATGTTCAAGAGCACTCCGTATAG	
PP2C.D3-FW1	CACCGAGACGCGTTTCCATCTCTC	1607 bp
PP2C.D3-RV1	AGTAGAAGGTCCAGCTAAATCAC	
PP2C.D4-FW1	CACCGCCATTAGTTCAAAAAGAGACTAGC	1036 bp
PP2C.D4-RV1	TAAAACGGGATTATGGGCTGAG	
PP2C.D5-FW1	CACCGGAACGTGTCTCGCTATTCC	3075 bp
PP2C.D5-RV1	GGAGGCGCCAGCAGCAGCAG	
PP2C.D6-FW1	CACCGACGTGGAGGGATGTTTGTT	3058 bp
PP2C.D6-RV1	GATTTTCTTGGGGAATGTCATAC	
PP2C.D7-FW1	CACCTTATTAACGGGCCATATATTGAAAA	648 bp
PP2C.D7-RV1	AAGTTTCTTAGGTAAAGTGATACC	
PP2C.D8-FW1	CACCTGATCAGATCTCGTTGACCAGT	2075 bp
PP2C.D8-RV1	GTATAACACATTGAGTAACCGTC	
PP2C.D9-FW1	CACCCTCAAAGCCCAGTTTGTGG	591 bp
PP2C.D9-RV1	AGAGAGGAAGATACTGAACTTGG	
	**RT-PCR Analysis of *pp2c.d* T-DNA lines**	
PP2C.D1-888F	CACAAGATCCATAGGTGATGC	
PP2C.D1-3’R	TGCCCTTGACACAGGAAGTG	

PP2C.D2-697F	GTATCGAGGTCTATAGGAGAC	
PP2C.D2-978R	CGCTGCCTCGTGAAGAGCCG	
PP2C.D5-amiF	CGCGGATGATTCTGGACTAT	
PP2C.D5-amiR	TCTGGCGAGTAAAAGGCATC	
PP2C.D6-49F	CGCTTTGACGGATGTGGAGG	
PP2C.D6-158R	GCTTGAACAACAGCCATGGAG	
PP2C.D7-amiF	CAATGTCTGGCGTGTTAAGG	
PP2C.D7-amiR	CATGGAAATGCCTCCTGACT	
PP2C.D8-amiF	GCCGTAGCTGAACGGTTATC	
PP2C.D8-amiR	CCAGAGACCATCTGATGCAA	
PP2C.D9-F2	GGATGTTCTCCTGGTTAGCGAG	
PP2C.D9-R2	GAAGCTTCAGGACCACCATGACC	
